# Supplementary material for: Pathogen subversion of neuro-epidermal signaling impairs lysosomal function to disrupt collagen homeostasis in Caenorhabditis elegans
Source: PLoS Biol. 2026 Sep 22;24(9):e3004016. doi: 10.1371/journal.pbio.3004016 (PMC13649122; doi:10.1371/journal.pbio.3004016)

Figure 2 L

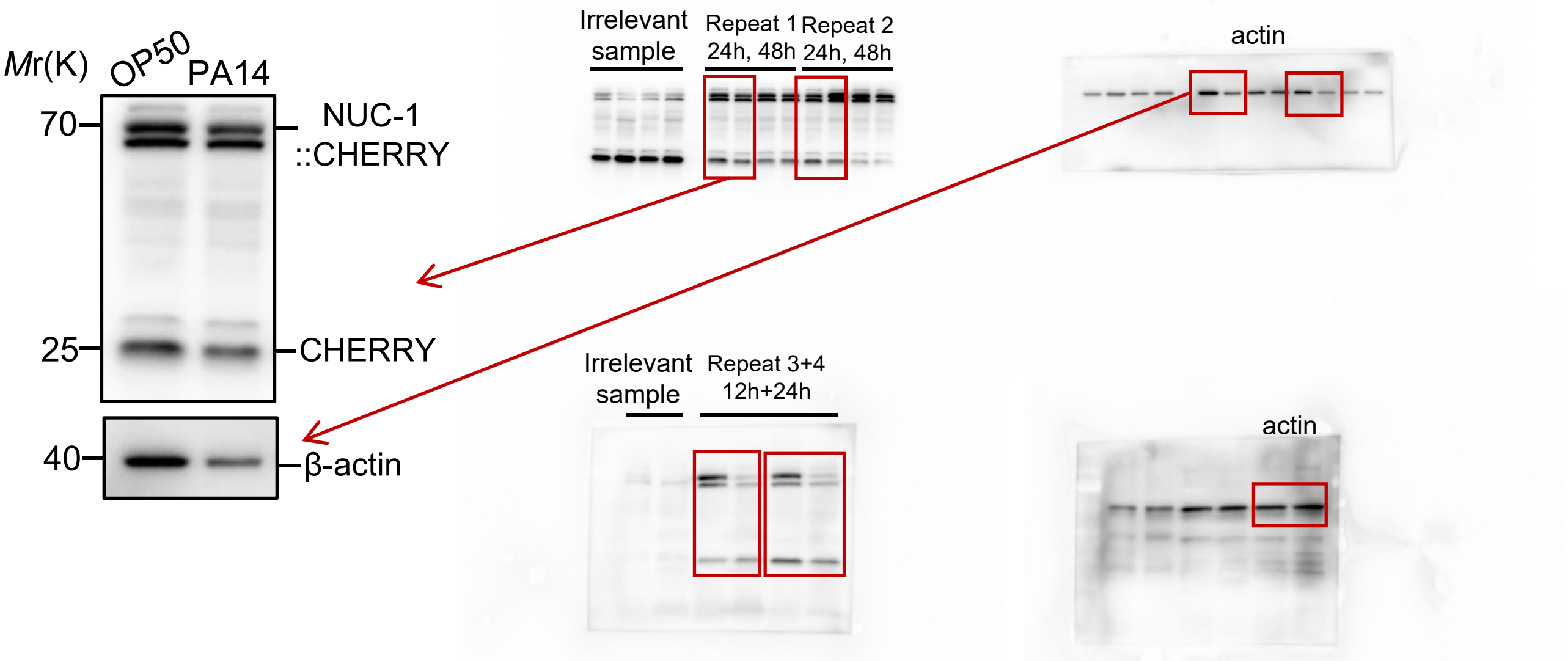

Figure 3B

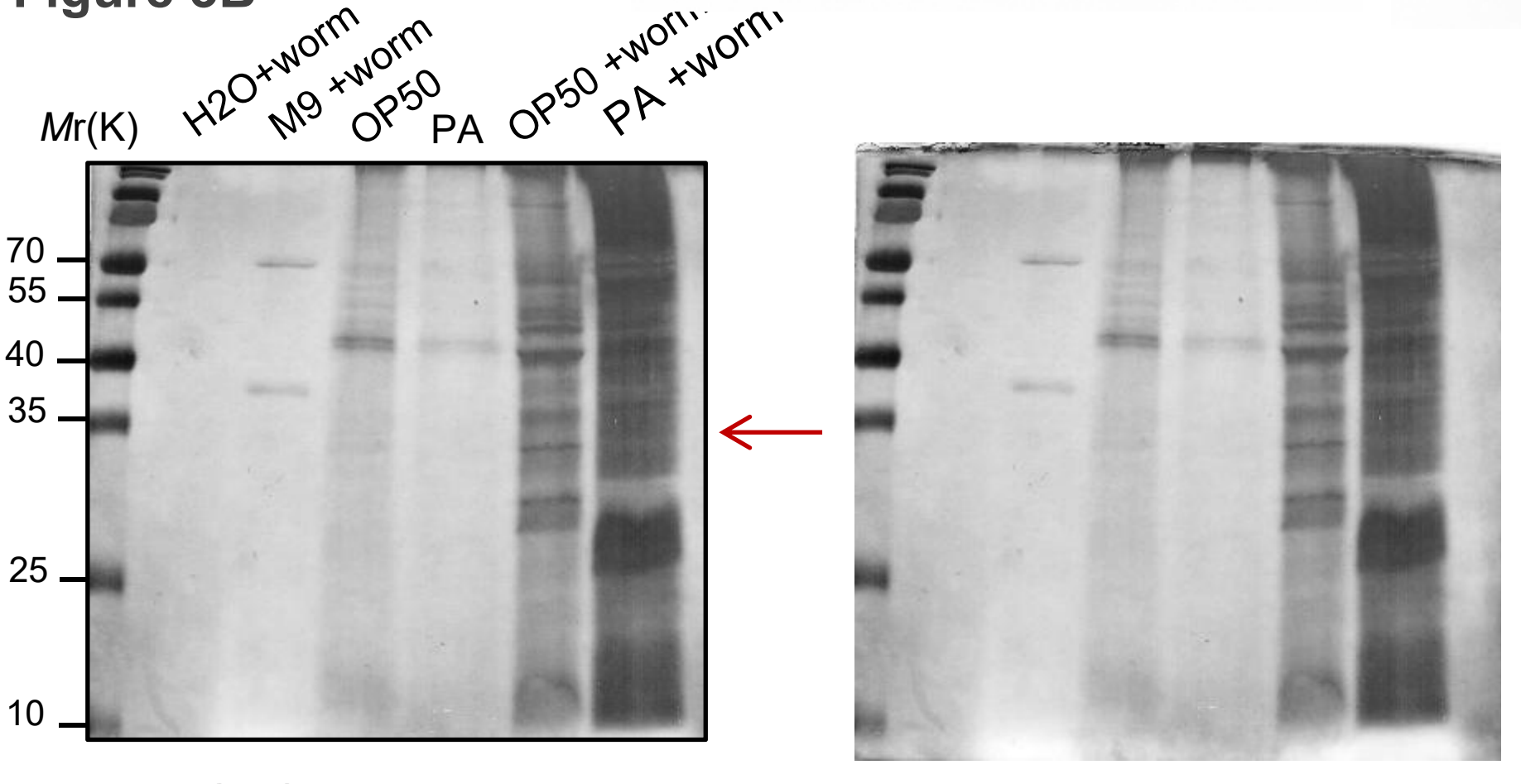

Figure S6C

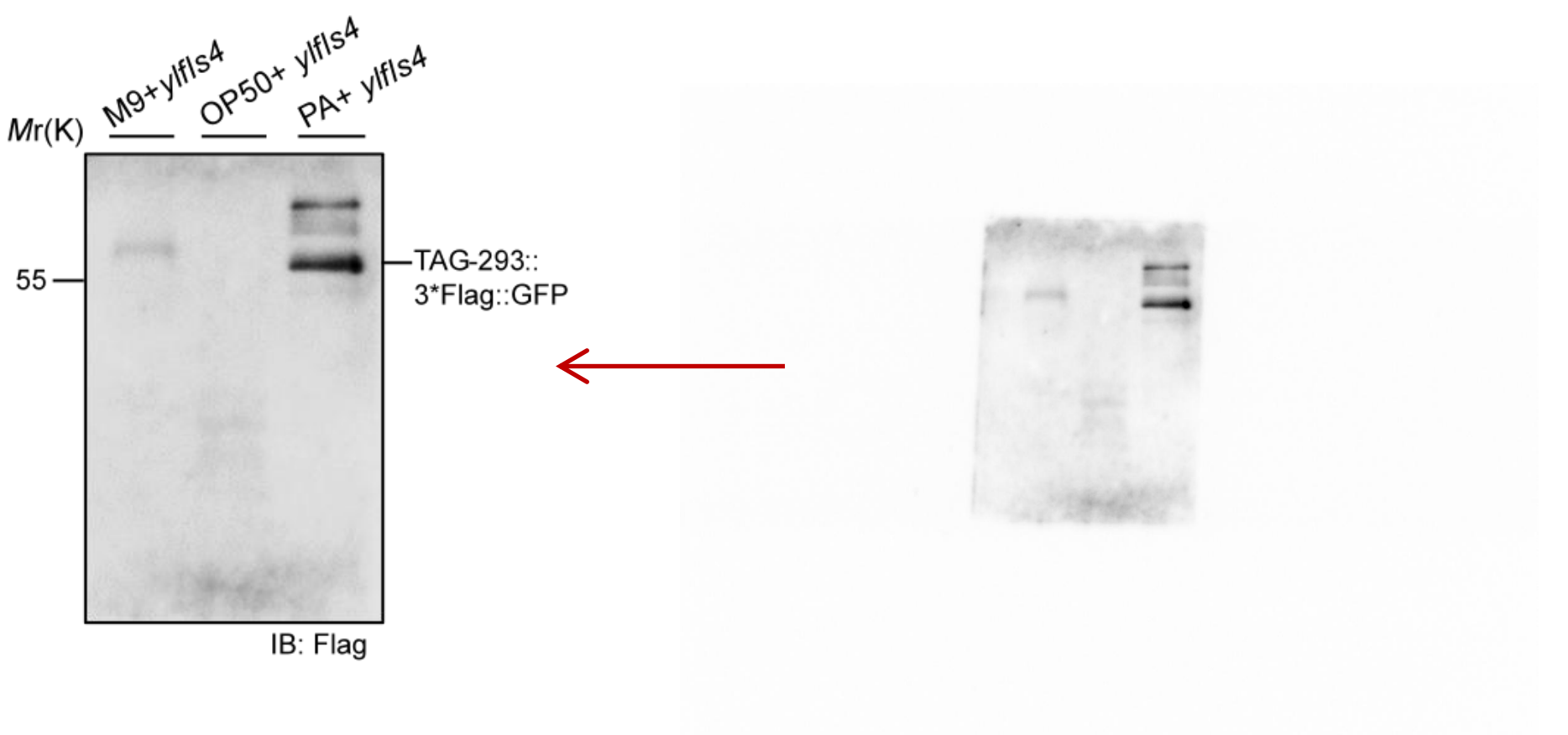

Figure 5D

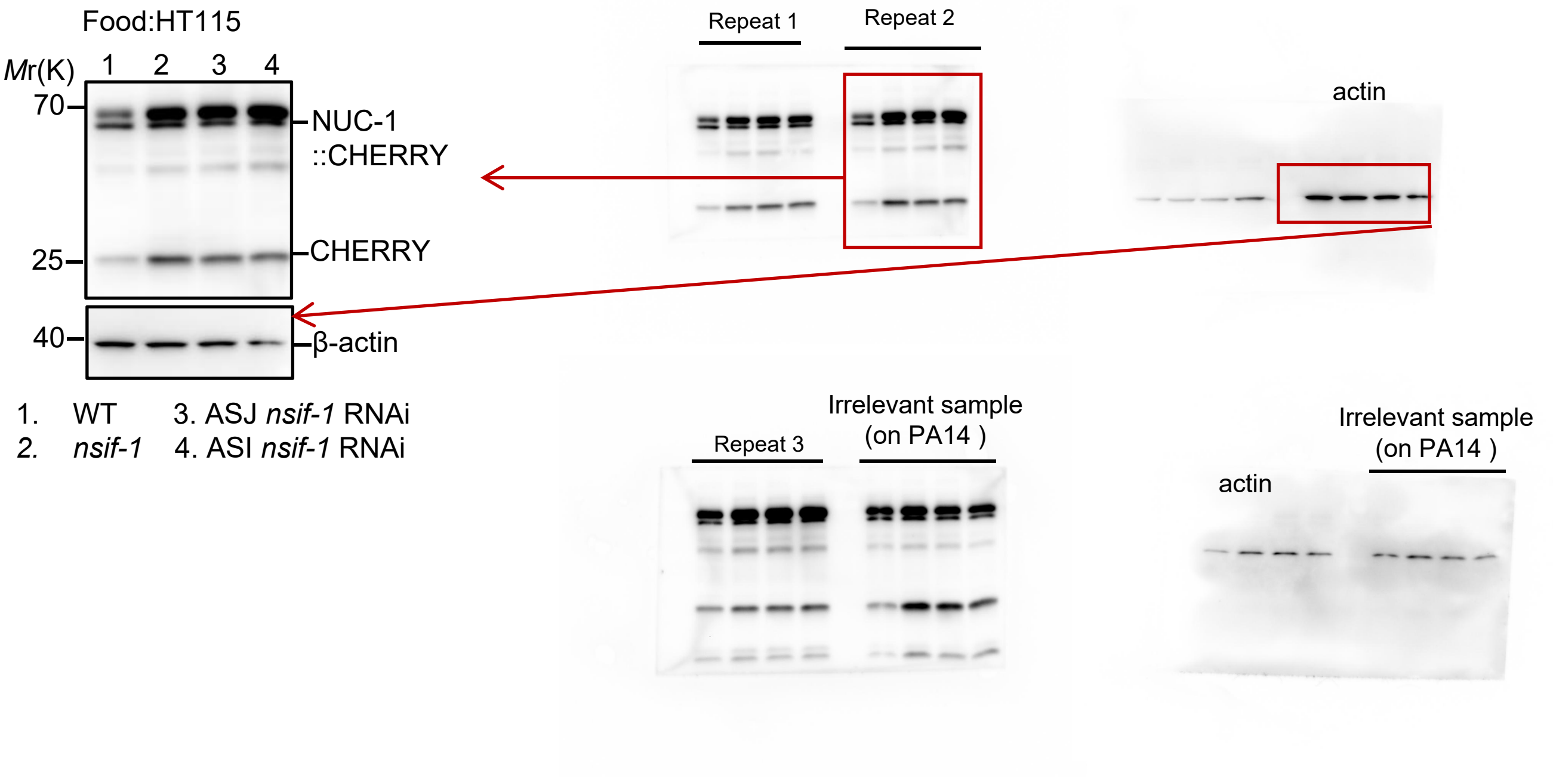

Figure 5E

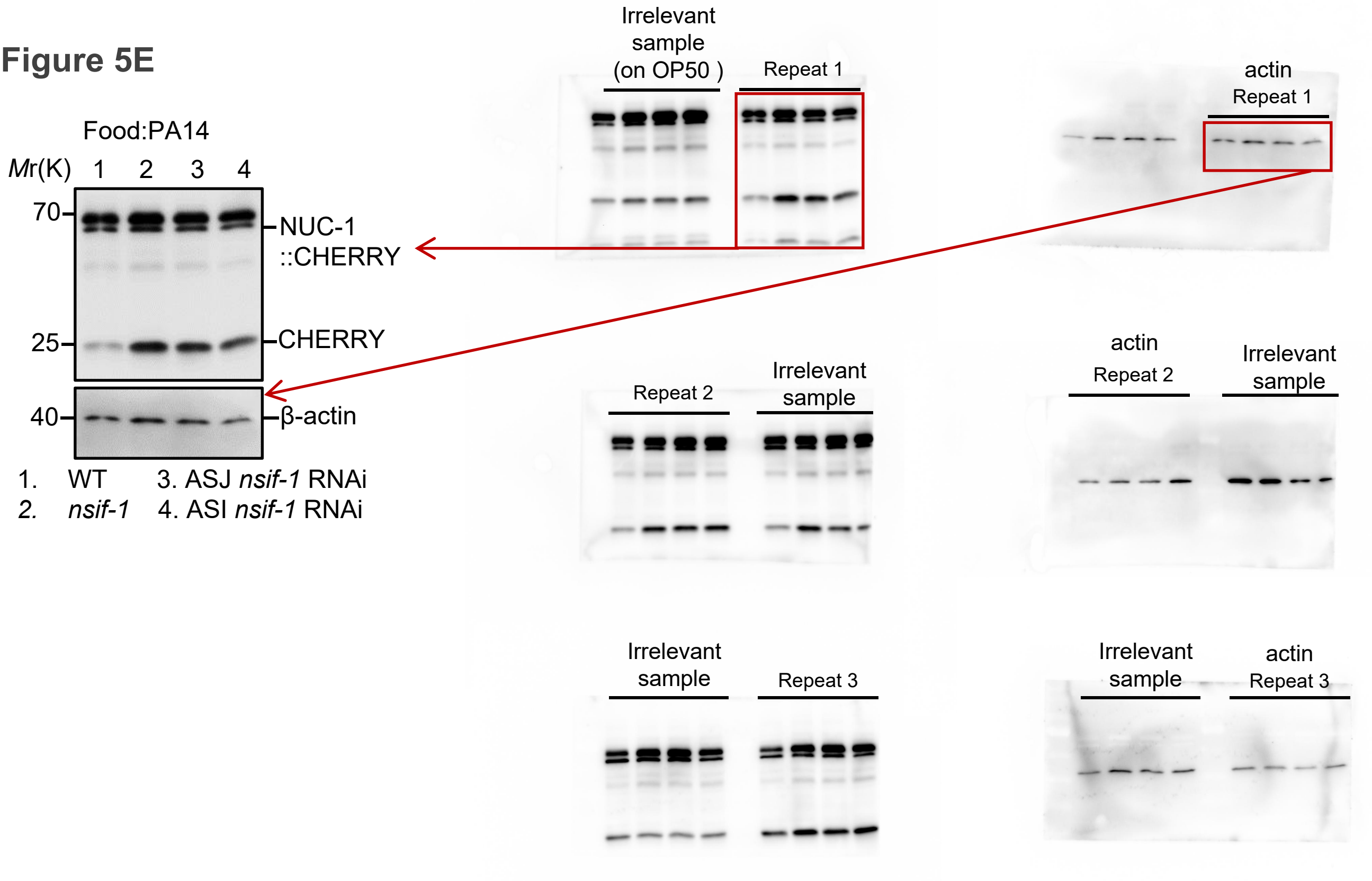

Figure 6E

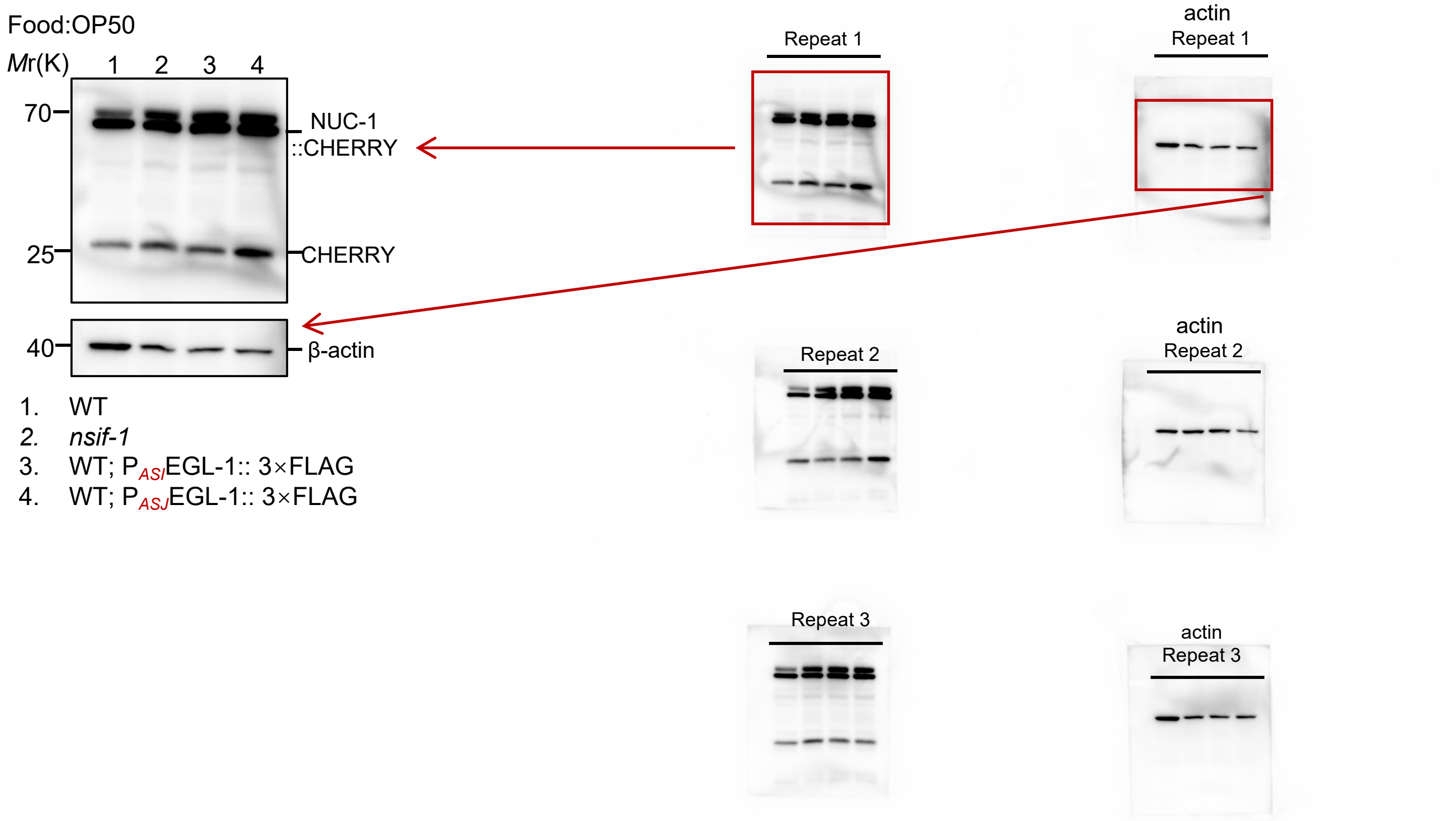

Figure 6F

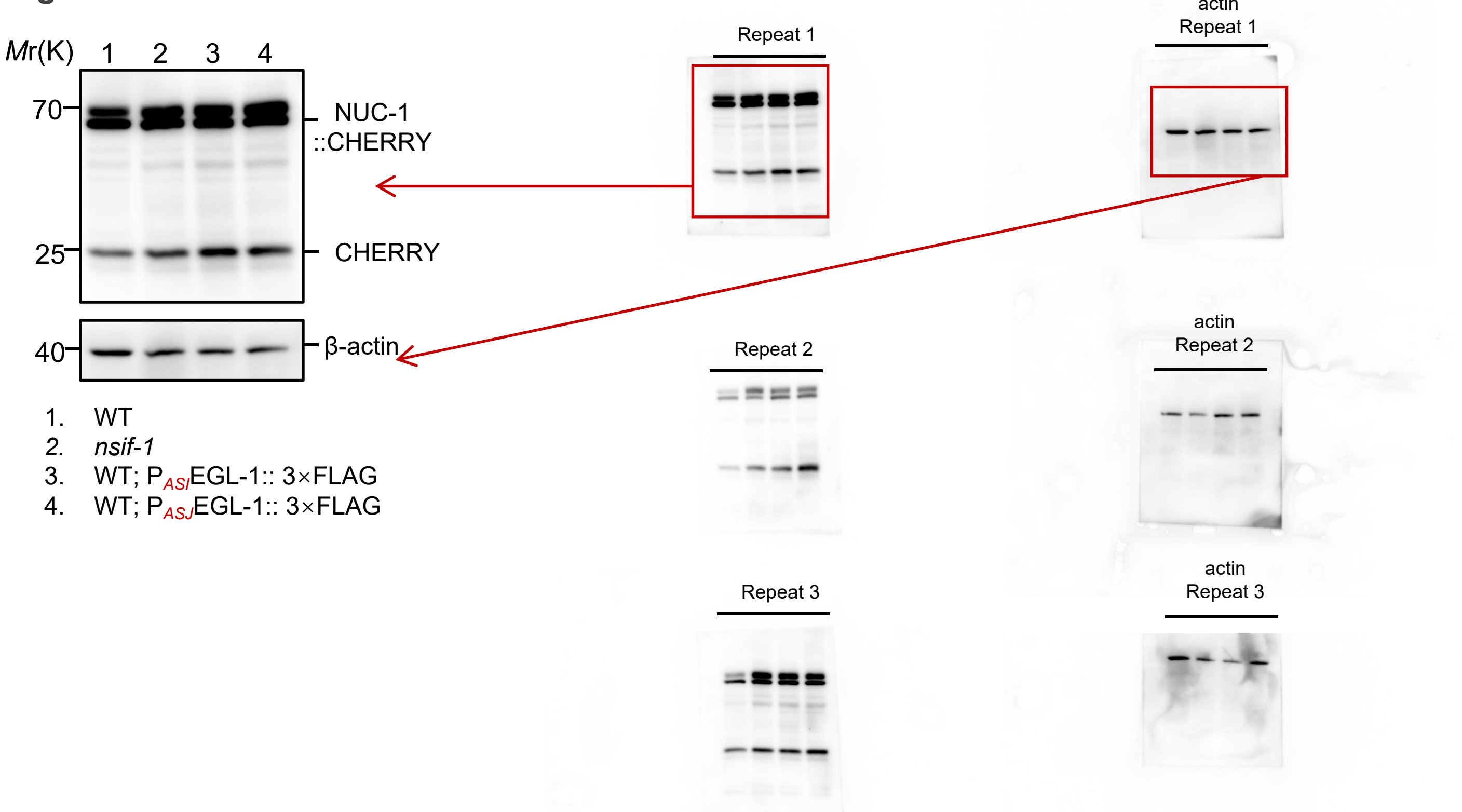

Figure 7D

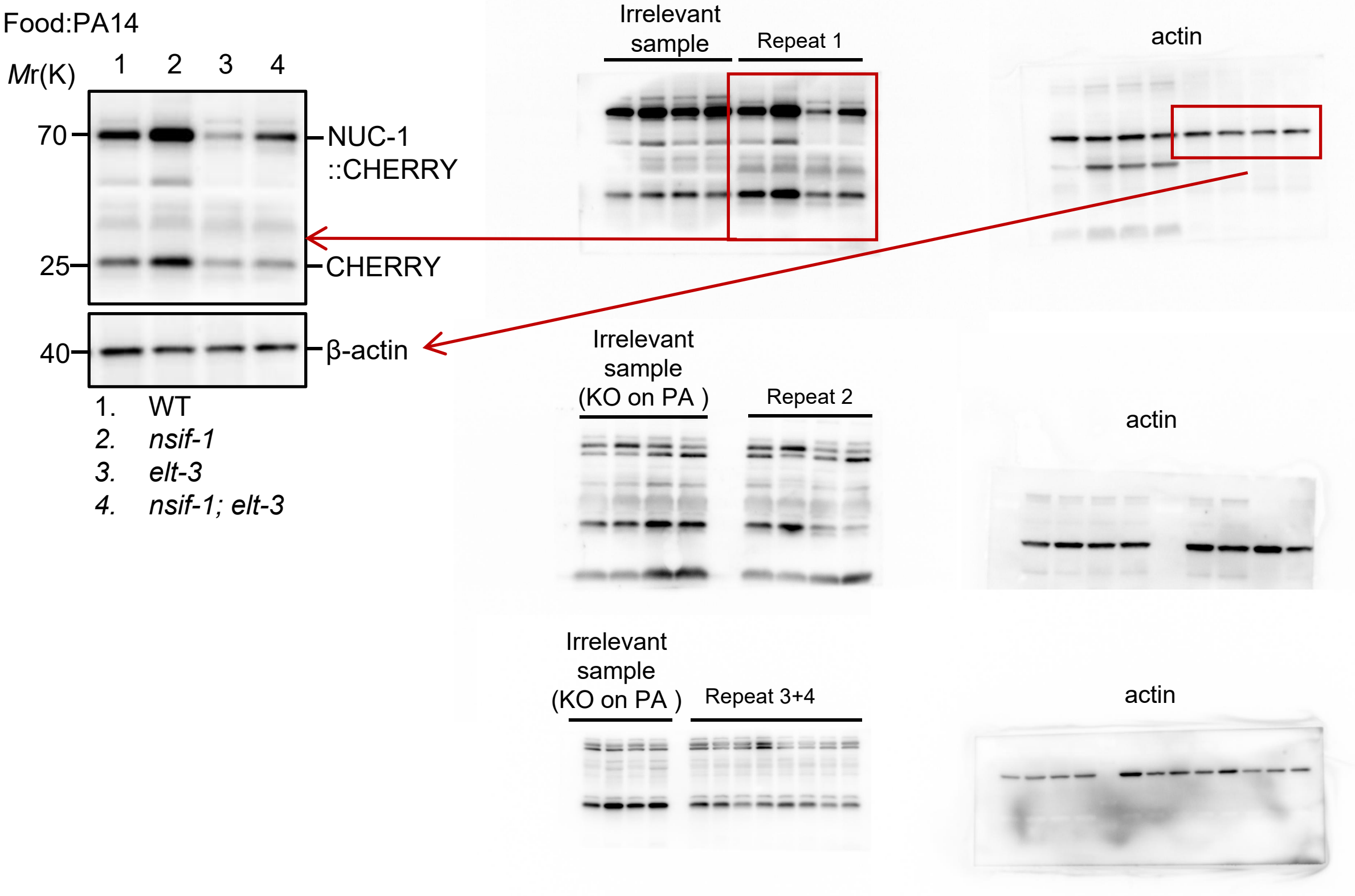

Figure S22F

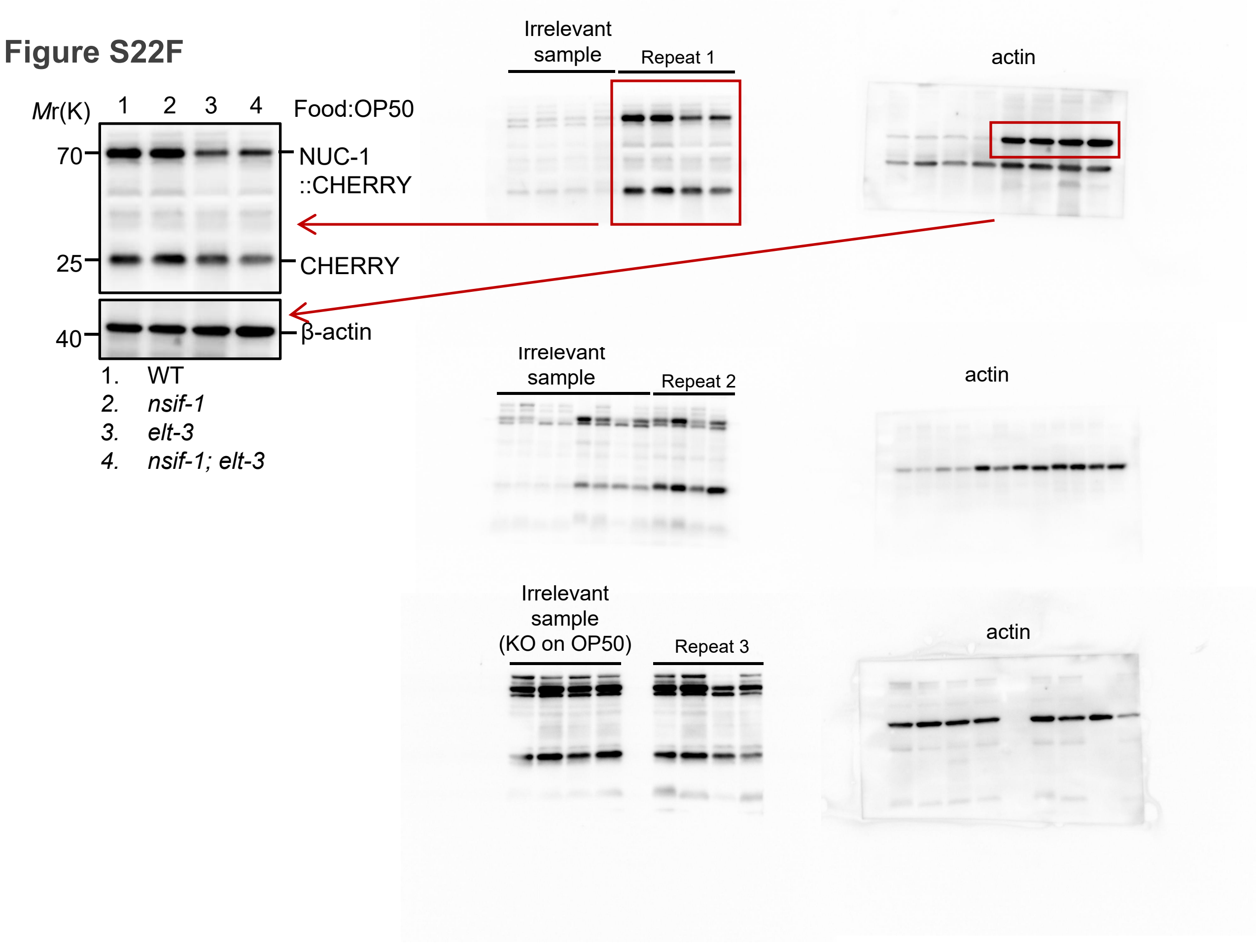

Supplement: Supplementary file 1 [file pbio.3004016.s029.pdf]
