# Supplementary material for: Pathogen subversion of neuro-epidermal signaling impairs lysosomal function to disrupt collagen homeostasis in Caenorhabditis elegans
Source: PLoS Biol. 2026 Sep 22;24(9):e3004016. doi: 10.1371/journal.pbio.3004016 (PMC13649122; doi:10.1371/journal.pbio.3004016)
Supplement: Supplementary file 2 [file pbio.3004016.s030.pdf]

Point-by-Point Response to Reviewers

Dear Editor and Reviewers,

We sincerely thank the editor and all reviewers for their careful evaluation and constructive comments on our manuscript. In this revision, we have performed the additional experiments requested, refined our text and figures, and addressed every point raised. New experimental data have been added to the manuscript file (All changes are highlighted in Red text). Reviewer comments are shown in blue text, followed by our responses in plain text.

Response to Reviewer #1

We thank Reviewer #1 for the supportive evaluation and for the constructive comment, which we address below.

Rev. 1:

I would support the publication, though my minor concern still remains as I did not find the control data after reading the long PDF file:

Minor Concerns

1. The study identifies 35 candidate proteins in the supernatant of PA14-infected worms using mass spectrometry, narrowing these to five neuronal secreted proteins based on SignalP-5.0 and CeNGEN data. However, the manuscript does not report whether proteins from control conditions (e.g., OP50-treated or M9 buffer) were similarly analyzed to confirm specificity to PA14 infection. Providing a comparative proteomic profile of control supernatants would strengthen the claim that NSIF-1 (TAG-293) is specifically induced by PA14. The authors should include mass spectrometrydata from control conditions or explicitly state why such controls were not feasible.

Response: We thank the reviewer for this important point. We did include the relevant control conditions in our experimental design.

As shown in Figure 3B (silver staining), we collected supernatants from five conditions in parallel: (1) worms in M9 buffer alone, (2) worms exposed to E. coli OP50, (3) worms exposed to P. aeruginosa PA14, (4) OP50 only (no worms), and (5) PA14 only (no worms). The bacteria-only conditions (4 and 5) served to exclude bacterial contributions to the secretome. Mass spectrometry was then performed on supernatants from worm-containing conditions (worms exposed to E. coli OP50 and PA14), and the 35 candidate proteins reported represent those that were specifically induced or significantly enriched in the PA14-exposed worm supernatant relative to OP50-exposed worm supernatant. The complete comparative dataset, including peptide intensities across conditions, is provided in S1 Table.

We have now revised the Results section to explicitly state that "Mass spectrometry identified 35 candidate proteins induced by PA14 (S6A Fig, S1 Table) ".

Response to Reviewer #2

We are grateful to Reviewer #2 for the positive overall assessment and for raising the additional cuticle time-course question, which was important for definitively establishing the model. We have performed the requested experiment and made the requested corrections.

Rev. 2:

The authors have done a very nice job responding to the 2nd set of critiques while under review at EMBO J. Reviewer 3's comments bring up one critical point that had been bothering me and can be addressed with one experiment that needs to be performed before publication. In the clarification of the timecourse, the authors argue that their data indicate that infection disrupts intact cuticles based on growing L4 animals on PA14 and imaging DPY-7::GFP and 0, 3, 6, 12, and 24 hours later. While their results are consistent with this model it still doesn't rule out an effect on building the cuticle that takes time to manifest. The authors should rule the confounding variable of molting by taking dpy-7::GFP young adults or L4+1 day animals and placing them on PA14 and imaging 0, 3, 6, 12, and 24 hours later. If they see the same results then it would definitively support their model and is a straightforward experiment to perform. Once that experiment is performed and the minor corrections listed below are addressed this manuscript will be suitable for publication in PLOS Biology.

Response:

We thank the reviewer for this excellent suggestion, which directly tests whether PA14-induced cuticle damage requires concurrent cuticle assembly. We performed the experiment exactly as proposed.

DPY-7::sfGFP young adults (L4 + 24 h, well past the L4-to-adult molt) were transferred to either OP50 (control) or PA14 lawns and imaged at 0, 3, 6, 12, and 24 hours after transfer. PA14-exposed adults exhibited a significant increase in animals with disorganized DPY-7 pattern as early as 3 h post-transfer, and the proportion of affected animals increased progressively at 6, 12, and 24 h, reaching highly significant differences from OP50 controls at every time point.

Because the animals were already post-molt young adults at the time of transfer and did not undergo any additional molt within the 24 h window, this experiment definitively rules out a molting/cuticle-assembly artifact and demonstrates that PA14 directly damages the mature, fully-

formed adult cuticle. This new data has been added to the manuscript and is described in the Results section (New data: S3 Fig).

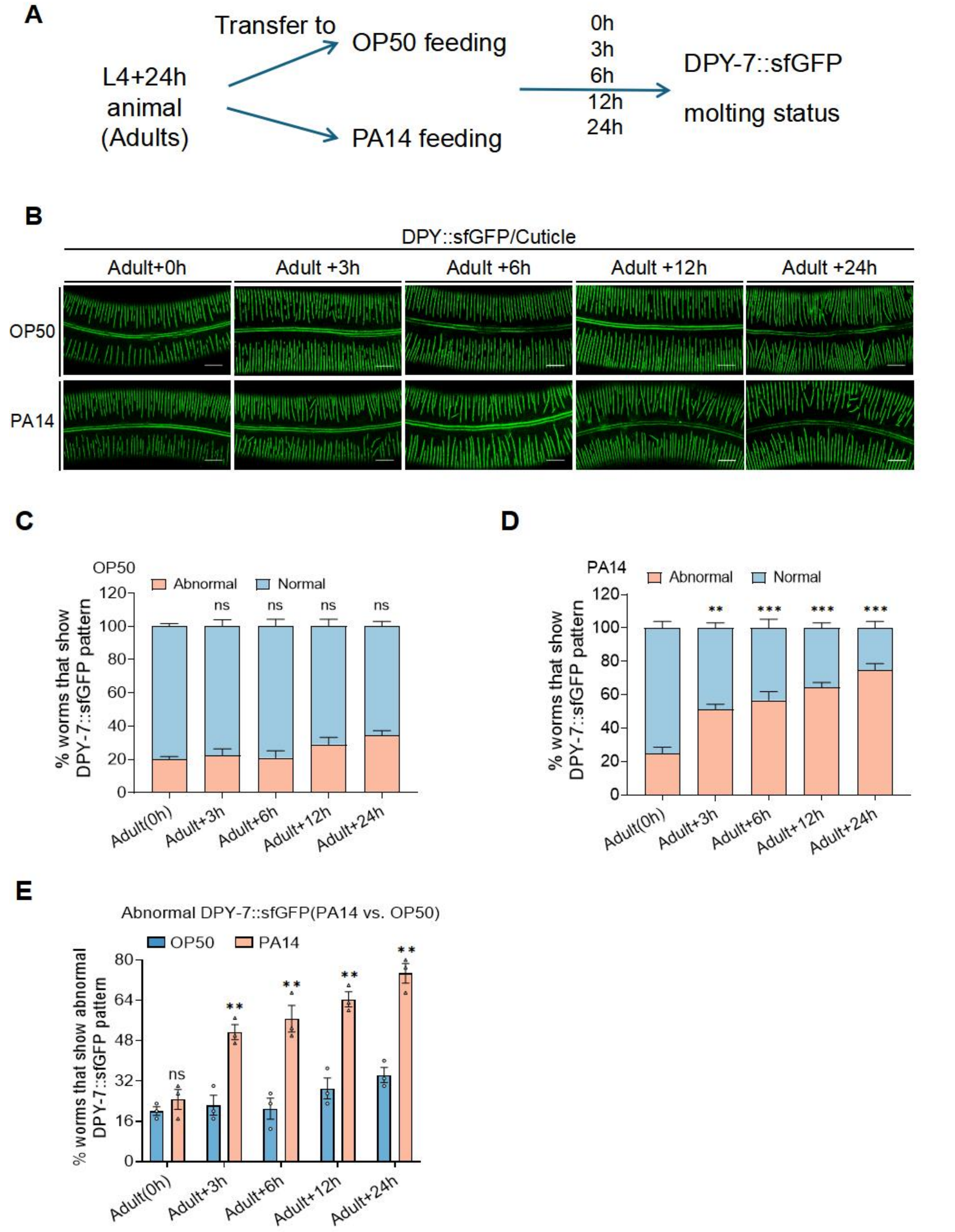

S3 Fig. PA14 induces cuticle damage during the adult stage.
(A) Schematic diagram of the timelapse microscopy method for observing cuticle morphological changes in wild type (WT)) animals from L4+24h(adult) stage upon PA14 infection. DPY-7::sfGFP was observed after transferring L4+24h(adult) stage

animals to OP50 or PA14 for 0, 3, 6, 12, 24 hours.
(B) Confocal microscopy images DPY-7::sfGFP of L4+24h(adult) animals after 0, 3, 6, 12, 24 hours OP50 or PA14 exposure. Scale bars: 10 µm.
(C-E) Quantitative analysis of the worms with abnormal DPY-7::sfGFP pattern on OP50(C) and PA14(D) in different stage. And quantitative analysis of the worms with abnormal DPY-7::sfGFP pattern percentage between OP50 and PA14 in the same stage(E). n=90 animals.
For all quantification, data are presented as mean ± SEM. Statistical comparisons were performed using one-way ANOVA followed by Tukey's multiple comparisons test (C, D). *$p<0.05$, **$p<0.01$, ***$p<0.001$, n.s., not significant. All experiments were performed independently at least three times. The data underlying this Figure can be found in S2 Data.

Minor points

1. The authors should add citations for the strains obtained from the CGC. Some of these are missing from the reference section. Go through the strains in that section on the CGC site and be sure to add the appropriate citations.

Response: We thank the reviewer for catching this. We have gone through every CGC strain listed in the Strains section and added the appropriate primary citations.

We cited reference for these strain:
XW5399: *qxIs257*[P_{ced-1}NUC-1::CHERRY] [75];
XW17096: *qxIs383*[P_{hyp-7}NUC-1::CHERRY] [18, 51];
XW13734: *qxIs612*[P_{hsp}NUC-1::sfGFP::CHERRY] [18, 51];
XW18042: *qxIs722*[P_{dpy-7}DPY-7::sfGFP, single-copy insertion] [18];
XW19180: *qxIs750* [P_{hsp}NUC-1::pHTomato] [18, 51];

18. Miao R, Li M, Zhang Q, Yang C, Wang X. An ECM-to-Nucleus Signaling Pathway Activates Lysosomes for C.Â elegans Larval Development. Dev Cell. 2020;52(1). doi: 10.1016/j.devcel.2019.10.020. PubMed PMID: 31735670; PubMed Central PMCID: PMCQ1.
51. Sun Y, Li M, Zhao D, Li X, Yang C, Wang X. Lysosome activity is modulated by multiple longevity pathways and is important for lifespan extension in C. elegans. Elife. 2020;9. doi: 10.7554/eLife.55745. PubMed PMID: 32482227; PubMed Central PMCID: PMCQ1.
75. Guo P, Hu T, Zhang J, Jiang S, Wang X. Sequential action of Caenorhabditis elegans Rab GTPases regulates phagolysosome formation during apoptotic cell degradation. Proc Natl Acad Sci U S A. 2010;107(42):18016-21. Epub 2010/10/06. doi: 10.1073/pnas.1008946107. PubMed PMID: 20921409; PubMed Central PMCID: PMCPMC2964220.

2. The tissue-specific knock-out methods need to be removed since those experiments are no

longer in the paper.

Response: Agreed. We have removed the section in Methods describing tissue-specific CRISPR/Cas9 knockout.

Response to Reviewer #4

We thank Reviewer #4 for the careful and detailed review. The reviewer's comments have prompted a substantial set of new experiments and clarifications that have meaningfully strengthened the manuscript. We address each point below.

Rev. 4:
Summary:
In this manuscript, the authors discover that PA14 infection disrupts cuticle structure and hypodermal lysosome function in C. elegans. They implicate nsif-1 as the mediator of this process by demonstrating that it is required for the deleterious effects of PA14 on cuticle formation and lysosomal function. The authors then demonstrate that nsif-1 also functions in the intestine to regulate pmk-1-dependent immune response to PA14. While the identification of a neuronal-hypodermal host defense pathway is significant, the paper has many scientific and experimental deficiencies that need to be addressed before publication. In addition, the writing of this paper indicates a lack of attention to detail that needs to be addressed. For example, in the strain list, some genotypes are listed that are not presented in the paper at all; the figure legends are overly concise and do not accurately describe the data; and the methods sections frequently lack experimental detail.

Major issues:
The evidence that NSIF-1 secreted from ASI/ASJ and functions in the hypodermis presented in the paper is not complete. While the data presented indicate that neuronal-expressed NSIF-1 likely functions non-autonomously in the hypodermis, additional work is needed for the authors to conclude that NSIF-1 from ASI/ASJ is secreted to and functions in the hypodermis.

1) Demonstrate that fluorescently tagged NSIF-1 expressed only in ASI or ASJ is detected in the hypodermis and its nuclei, and that this is abolished when the SP of NSIF-1 is removed.

Response:

We thank the reviewer for this important request. We generated four new transgenic lines that express full-length NSIF-1::GFP or signal-peptide-deleted ΔSP NSIF-1::GFP under either the ASI-specific (*str-3*) or ASJ-specific (*trx-1*) promoter, each in a background carrying the hypodermal GFP-binding nanobody capture system (P*col-12*::GBP::mKate2). Because GBP::mKate2 traps any GFP that reaches the hypodermis, this system reads out tissue-to-tissue transfer with high sensitivity.

With full-length NSIF-1 expressed only in ASI or only in ASJ, we detected robust GBP-trapped GFP signal in the hypodermis. In contrast, ASI- or ASJ-driven ΔSP NSIF-1::GFP produced little to no hypodermal GFP signal under the same imaging conditions, indicating that the signal peptide is required for neuron-to-hypodermis transfer. These results directly demonstrate that NSIF-1 produced specifically in ASI or ASJ neurons is secreted and taken up by the hypodermis, and that this trans-tissue transfer requires the predicted secretion signal peptide. The new data have been added to the manuscript (New data: **Figure 4F**).

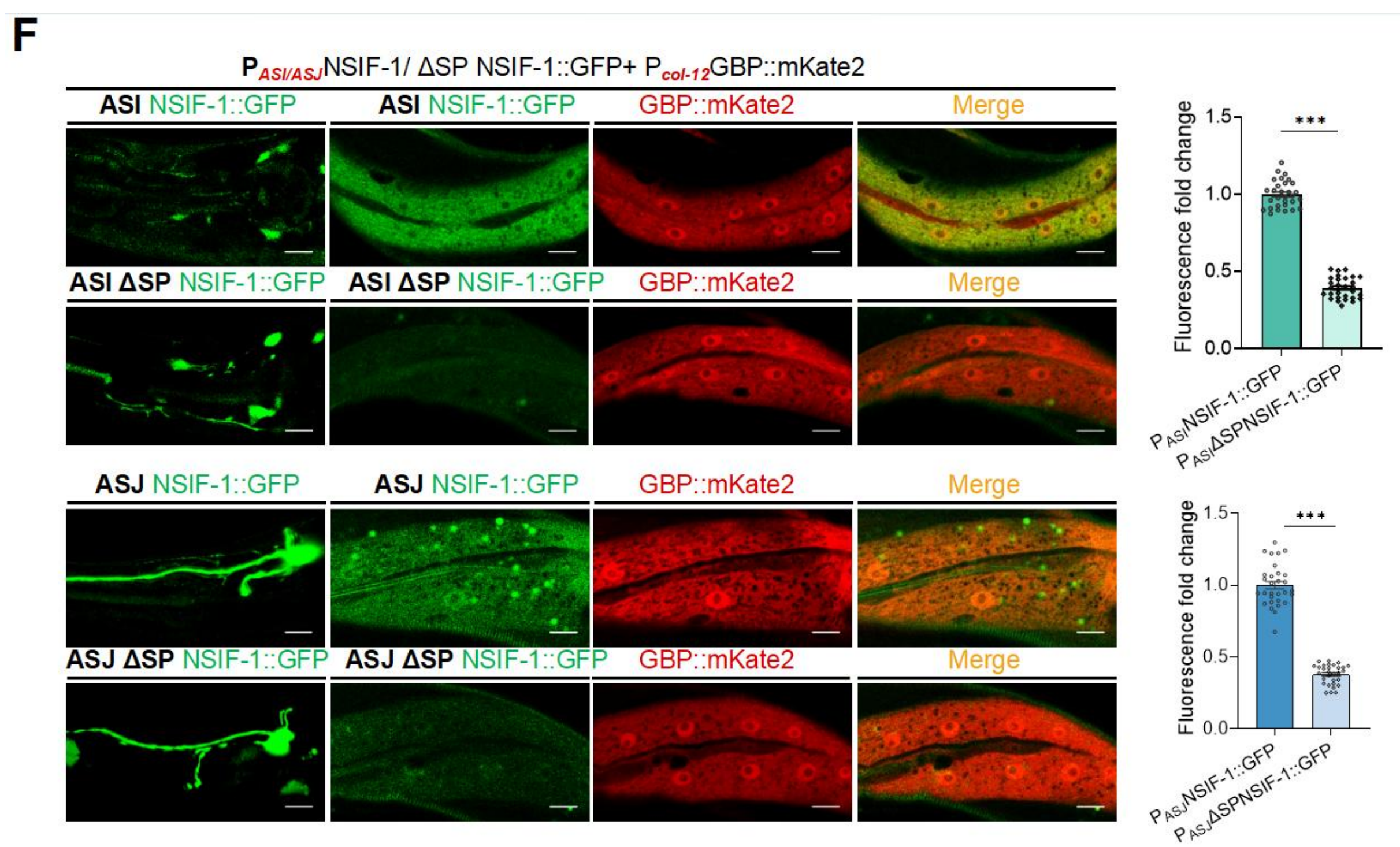

Figure 4 (F) Confocal fluorescence imaging of the transgenic line expressing NSIF-1::GFP and ΔSPNSIF-1::GFP in ASI(P_{str-3})/ASJ(P_{trx-1}) and GBP::mKate2 in epidermis(P_{col-12}). Expression of ΔSPNSIF-1 in ASJ and ASI neurons abolished the secretion of NSIF-1::GFP into the epidermis. Quantification of epidermal NSIF-1::GFP fluorescence intensity after PA14 exposure is shown (n=30 animals). Scale bar:10μm.

2) Demonstrate that NSIF-1 expressed only in ASI or ASJ rescues the cuticle and hypodermal lysosomal phenotypes of the nsif-1 mutant after PA14 infection and that this rescue is abolished

when the SP of NSIF-1 is removed.

Response: To ensure that our findings reflect the physiological role of NSIF-1, we performed comprehensive rescue experiments in the *nsif-1(ok1337)* mutant background. We expressed the NSIF-1 coding sequence under its native promoter, as well as under ASJ- (*trx-1*) or ASI-(*str-3*) specific promoters. Under PA14 infection, both the native and neuron-specific expression restored wild-type lysosomal morphology and rescued cuticle organization in the mutant (New data: Fig 6C-6D). These results support our model that neuronal secreted NSIF-1 acts non-cell-autonomously to regulate lysosomal function in peripheral tissues.

In our data, under PA14 infection, worms with ASI/ASJ-specific *nsif-1* knockdown maintained healthier lysosomal morphology, similar to that observed in *nsif-1(ok1337)* mutants (Fig 6A-6B), indicating that ASI/ASJ-derived NSIF-1 influences epidermal lysosomal function. However, we also examined versions lacking the signal peptide (ΔSP). Unexpectedly, ΔSP NSIF-1 expressed from ASI- or ASJ-specific promoters still rescued lysosomal morphology and cuticle organization in the mutant (New data: S17A-S17B Fig). One plausible explanation is that the elevated level of ΔSP NSIF-1 in ASI or ASJ may trigger other unknown signaling pathways that restore wild-type lysosomal morphology and rescue cuticle organization in the mutant.

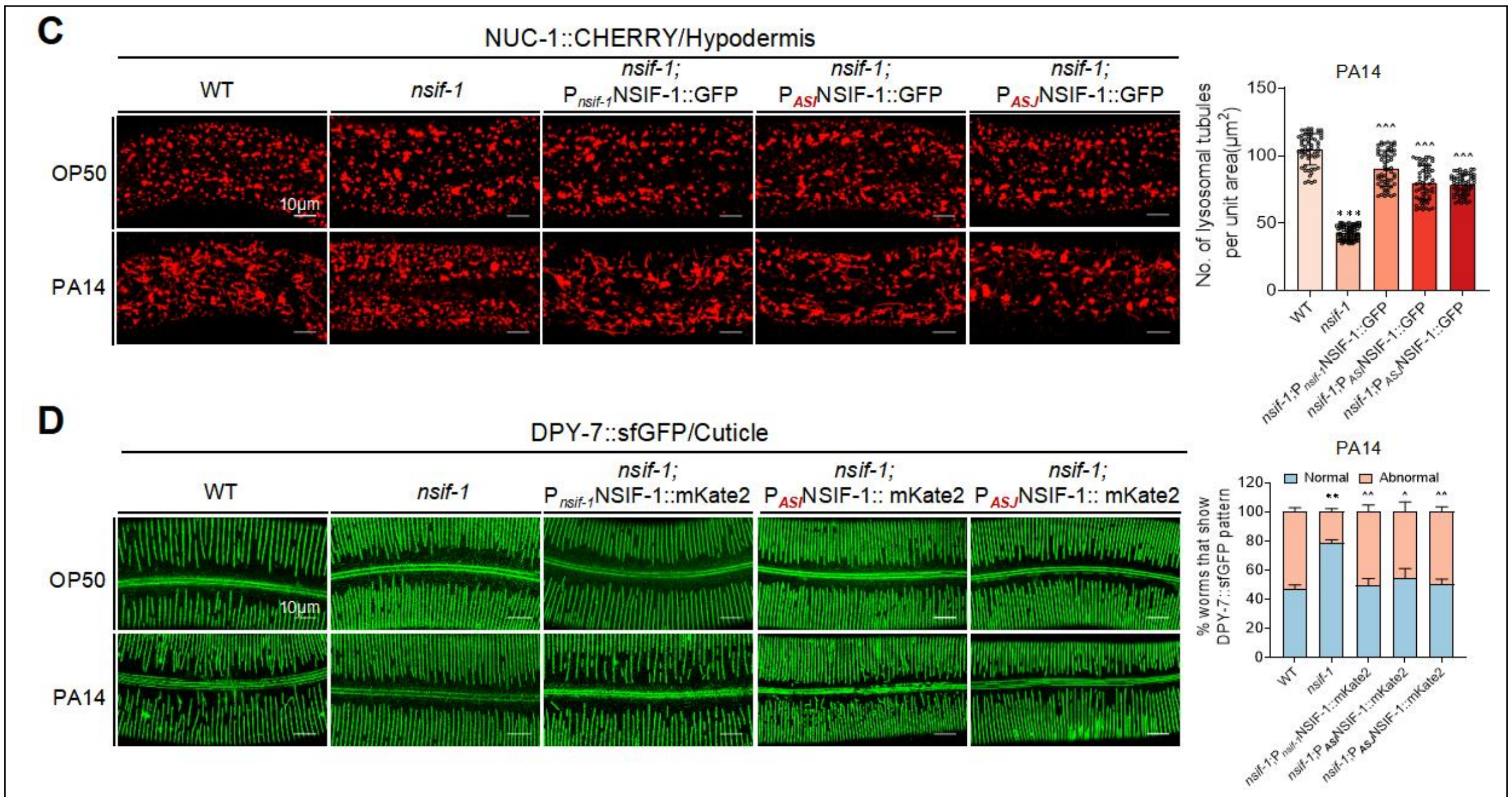

Figure 6 (C) Confocal fluorescence imaging and quantitative analysis of NUC-1::CHERRY marked lysosome in wild-type(WT), *nsfi-1(ok1337)*, *nsif-1(ok1337)*;P_{nsif-1}NSIF-1::GFP, *nsif-1(ok1337)*;P_{ASI}NSIF-1::GFP, *nsif-1(ok1337)*;P_{ASJ}NSIF-1::GFP animals following 24-h PA14 exposure (infection initiated at the L4 stage). Quantification of tubular lysosomes were quantified by counting three 35 × 25 µm² regions per worm (n = 60 worms) a total of 60 worms analyzed (n=60 animals). Scale bars: 10 µm.
(D) Confocal fluorescence imaging and quantitative analysis of DPY-7::sfGFP marked cuticle in wild-type (WT), *nsfi-1(ok1337)*, *nsif-1(ok1337)*;P_{nsif-1}NSIF-1::mKate2, *nsif-*

1(ok1337);P*ASI*NSIF-1::mKate2, *nsif-1(ok1337)*;P*ASJ*NSIF-1::mKate2 animals following 24-h PA14 exposure (infection initiated at the L4 stage). Quantification of abnormal DPY-7 pattern performed on 300 worms (n=300 animals). Scale bars: 10 μm.

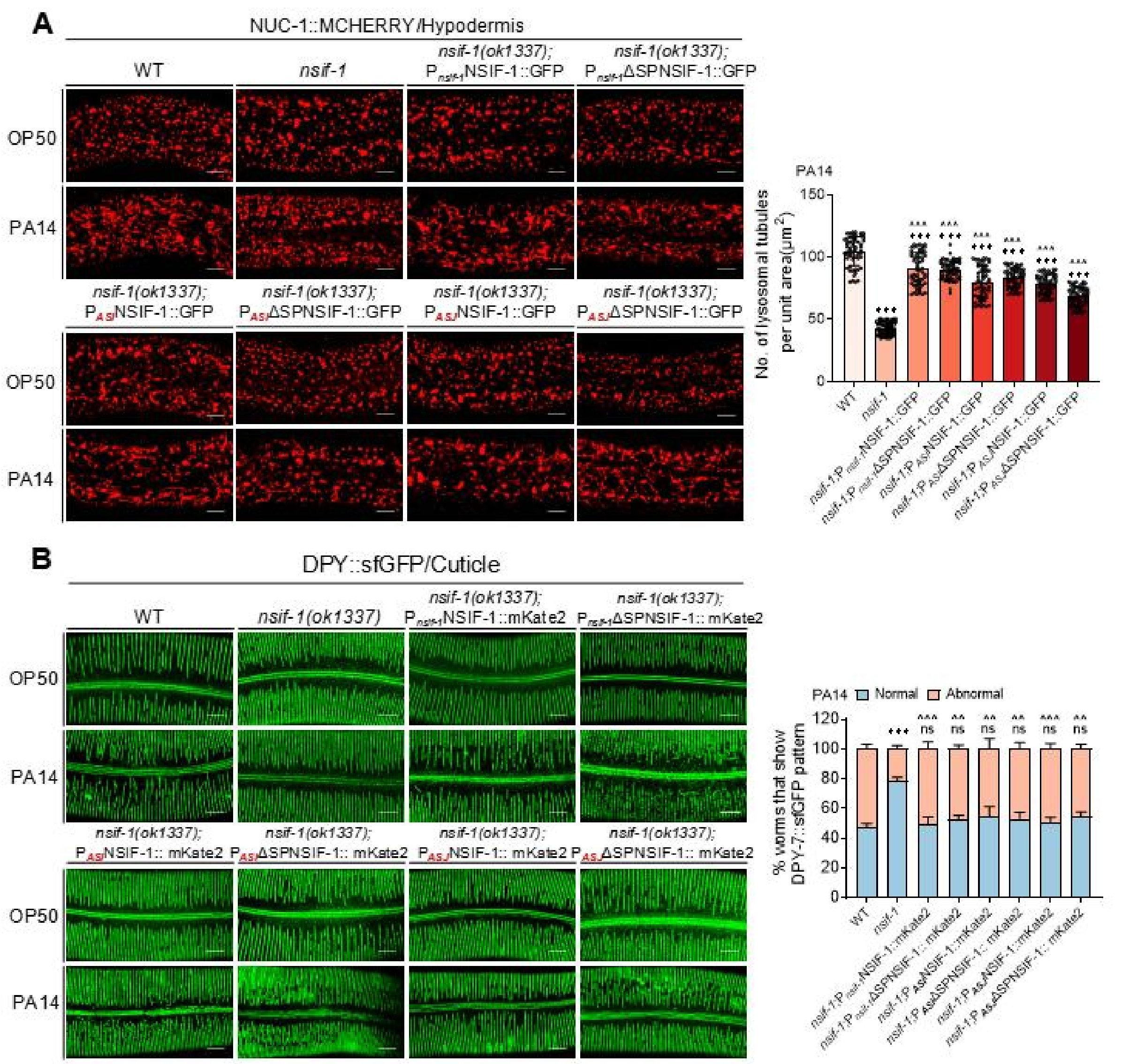

S17 Fig. Neuronal NSIF-1 in ASI/ASJ neurons is sufficient to impact lysosomal homeostasis and cuticle integrity.

(A) Confocal fluorescence images and quantitative analysis of NUC-1::mCherry-labeled lysosomes in wild-type (WT), *nsif-1(ok1337)* mutants, and *nsif-1(ok1337)* mutants expressing full-length NSIF-1::GFP or the signal-peptide-deleted variant (ΔSP-NSIF-1::GFP) under the control of the endogenous *nsif-1* promoter (P*nsif-1*), the ASI promoter (P_{ASI}), or the ASJ promoter (P_{ASJ}) following 24 h of PA14 exposure (initiated at the L4 larval stage). Quantification of tubular lysosomes were quantified by counting three 35 × 25 μm² regions per worm (n = 60 worms) . Scale bars: 10 μm.

(B) Confocal fluorescence imaging and quantitative analysis of DPY-7::sfGFP marked cuticle

in wild-type (WT), *nsif-1(ok1337)* mutants, and *nsif-1(ok1337)* mutants expressing full-length NSIF-1::GFP or the signal-peptide-deleted variant (ΔSP-NSIF-1::GFP) under the control of the endogenous *nsif-1* promoter (P*nsif-1*), the ASI promoter (P_{ASI}), or the ASJ promoter (P_{ASJ}) following 24 h of PA14 exposure (initiated at the L4 larval stage).. Quantification of abnormal DPY-7::sfGFP pattern performed on 300 worms. Scale bars: 10 μm.

3) Show that the expression from other tissues (muscle or intestine) does not rescue the nsif-1 mutant phenotypes when infected with PA14.

Response:

To test tissue specificity, we expressed NSIF-1 in the *nsif-1(ok1337)* background under the muscle-specific *myo-3* promoter or the intestinal-specific *vha-6* promoter. Neither muscle- nor intestine-driven NSIF-1 reduced the cuticle integrity benefit (DPY-7::sfGFP organization) or the healthier lysosomal morphology that characterizes *nsif-1(ok1337)* under PA14 infection (New data: S16A-B Fig). In other words, NSIF-1 supplied from these non-target tissues failed to reproduce wild-type NSIF-1 activity on hypodermal lysosomes and cuticle, consistent with the hypodermis being the relevant site of NSIF-1 action and with ASI/ASJ being the relevant source.

We have added this new data in the Results section of "Neuronal secreted NSIF-1 regulates epidermal lysosomes".

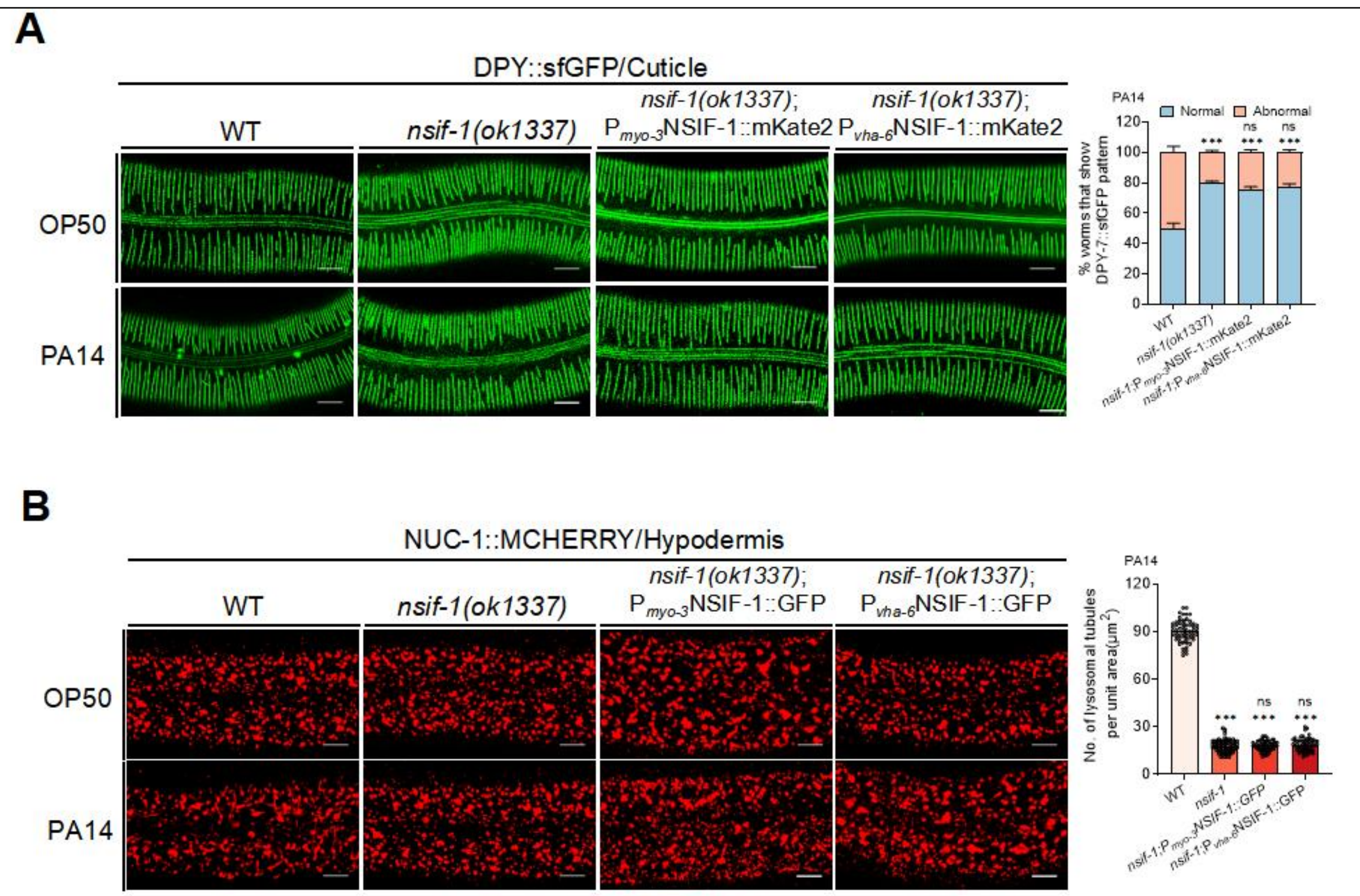

S16 Fig (A) Confocal fluorescence imaging and quantitative analysis of DPY-7::sfGFP marked cuticle in wild-type(WT), *nsfi-1(ok1337)*, *nsfi-1(ok1337)*;P*myo-3*::NSIF-1::mKate2, *nsfi-1(ok1337)*;P*vha-6*::NSIF-1::mKate2 animals following 24-h PA14 exposure (infection initiated at the L4 stage). Quantification of abnormal DPY-7::sfGFP pattern performed on 300 worms (n=300 animals). Scale bars: 10 µm.
(B) Confocal fluorescence imaging and quantitative analysis of NUC-1::CHERRY marked lysosome in wild-type(WT), *nsfi-1(ok1337)*, *nsfi-1(ok1337)*;P*myo-3*::NSIF-1::mKate2, *nsfi-1(ok1337)*;P*vha-6*::NSIF-1::mKate2 animals following 24-h PA14 exposure (infection initiated at the L4 stage). Quantification of tubular lysosomes were quantified by counting three 35 × 25 µm² regions per worm (n = 60 worms) . Scale bars: 10 µm.

4) Demonstrate that NSIF-1 expressed in the hypodermis rescues the nsif-1 mutant phenotypes when infected with PA14.

Response:

We expressed full-length NSIF-1::GFP and ΔSP NSIF-1::GFP under the hypodermis-specific *col-12* promoter in the *nsif-1(ok1337)* background. Both constructs disrupted the protective lysosomal and cuticle phenotypes of *nsif-1(ok1337)* even under OP50 feeding, and under PA14 infection the *col-12*-driven constructs eliminated the cuticle-protection and lysosome-protection benefits seen in the mutant. Notably, ΔSP NSIF-1 was as effective as full-length NSIF-1 in disrupting the hypodermis when expressed locally, consistent with NSIF-1 acting from within hypodermal cells once it arrives there (New data: S16C-D Fig).

These results confirm that NSIF-1 functions in the hypodermis to impair lysosomes and cuticle organization. We have added this new data in the Results section of “Neuronal secreted NSIF-1 regulates epidermal lysosomes”.

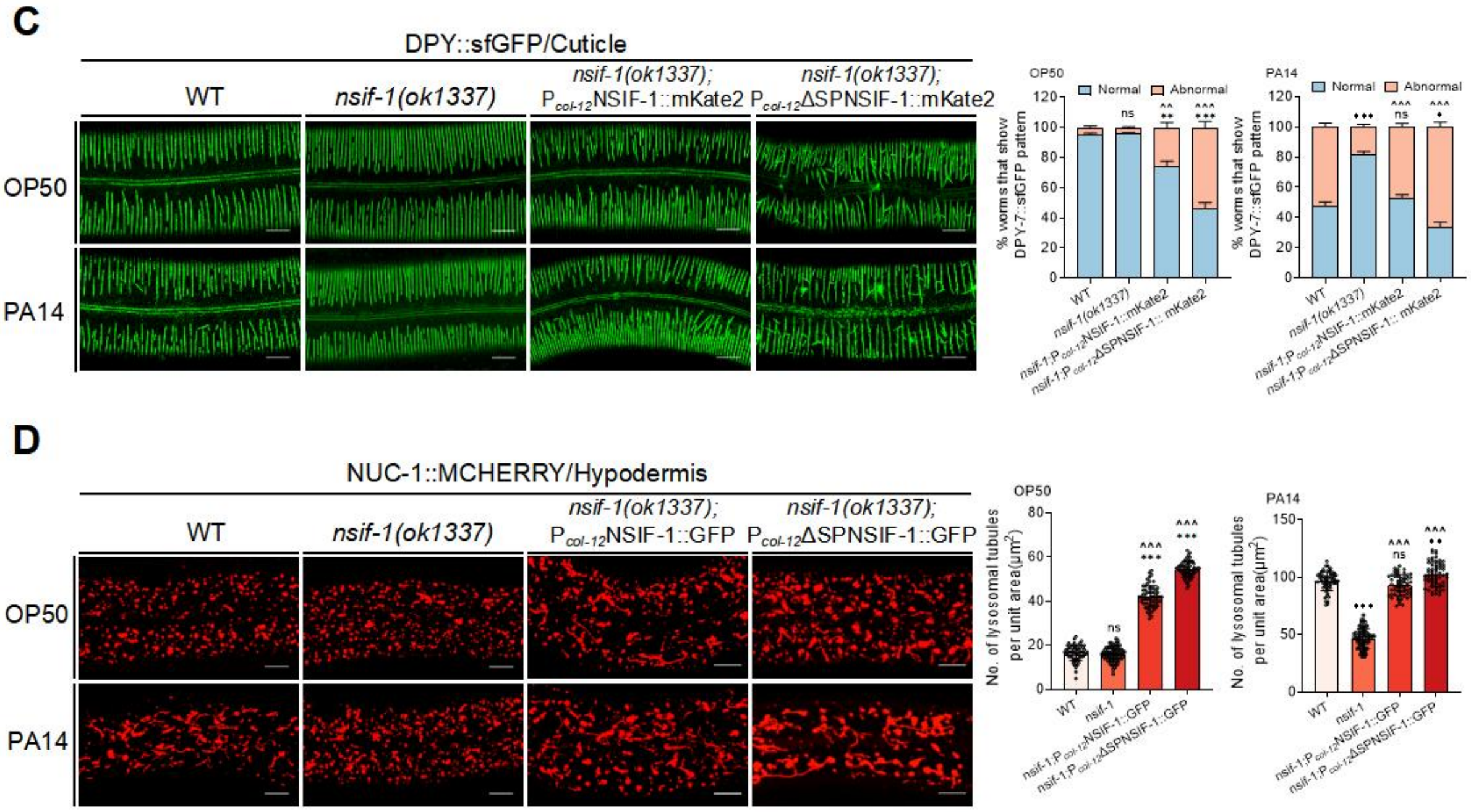

S16 Fig (C) Confocal fluorescence imaging and quantitative analysis of DPY-7::sfGFP marked cuticle in wild-type(WT), *nsfi-1(ok1337)*, *nsfi-1(ok1337)*;P*col-12*::NSIF-1::mKate2, *nsfi-1(ok1337)*;P*col-12*:: ΔSPNSIF-1::mKate2 animals following 24-h PA14 exposure (infection initiated at the L4 stage). Quantification of abnormal DPY-7::sfGFP pattern performed on 300 worms (n=300 animals). Scale bars: 10 µm.

(D) Confocal fluorescence imaging and quantitative analysis of NUC-1::CHERRY marked lysosome in wild-type(WT), *nsfi-1(ok1337)*, *nsfi-1(ok1337)*;P*col-12*:NSIF-1::mKate2, *nsfi-1(ok1337)*;P*col-12*:: ΔSPNSIF-1::mKate2 animals following 24-h PA14 exposure (infection initiated at the L4 stage). Quantification of tubular lysosomes were quantified by counting three 35 × 25 µm² regions per worm (n = 60 worms). Scale bars: 10 µm.

Issues in specific figures:
EV1B: How are "head" and "tail" defined? Landmarks? Size and position of a box?

Response:

We apologize for not providing sufficient detail in the original manuscript. The head and tail regions were identified based on characteristic morphological and anatomical landmarks. Specifically, the head was defined as the broader, rounded anterior end of the animal and was readily recognized by the autofluorescence of the pharynx. The tail was defined as the tapered posterior end and was identified by the autofluorescence near the anal opening (Response Figure-1). All confocal images were acquired at a resolution of 1024 × 1024. For fluorescence quantification, identical ROIs (Region of Interest) (78 µm × 60 µm) were positioned in the head and tail regions using these anatomical landmarks and applied consistently across all animals analyzed. This information has been added to the Methods section- “Hoechst Staining Assay”.

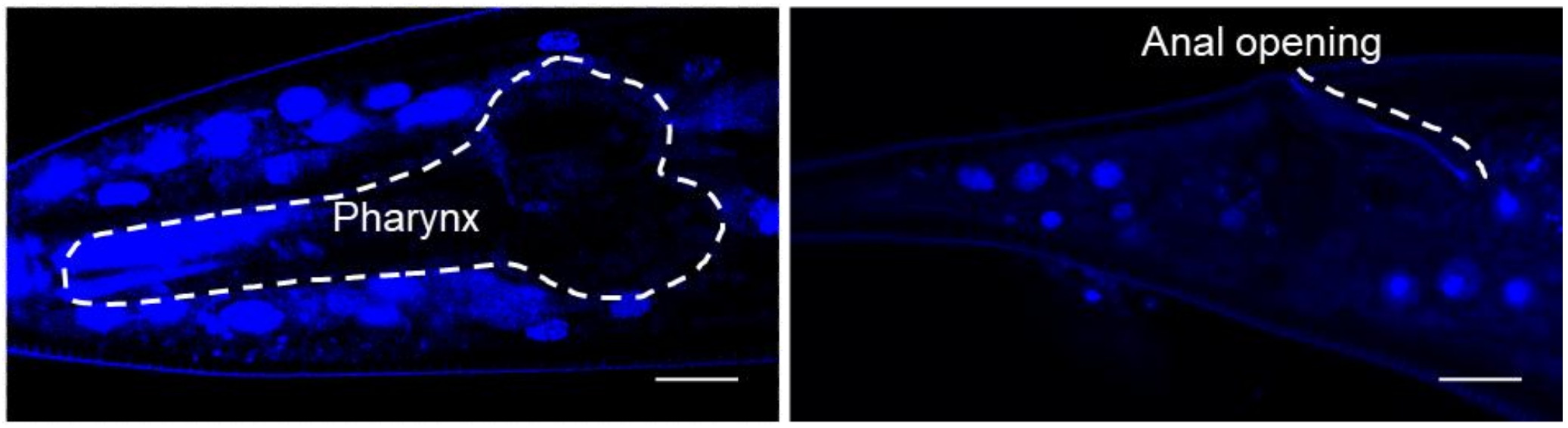

Response Figure-1. The image illustrates how we define the head and tail regions of animals.

S3: Fold change for each gene in each biological replicate should be plotted.

Response:

We have updated this figure to plot the fold change for each gene from each individual biological replicate (with individual data points overlaid on the bar plots). We now move this figure to **Figure 1D**.

2A,2F and 2I: How are the locations of the 35umx25um boxes determined. How do you ensure that they are in the same place in every worm?

Response:

We apologize for not providing sufficient detail in the original manuscript. All images were acquired using a Zeiss LSM 900 confocal microscope with a 63× oil-immersion objective at a resolution of 1024 × 1024 pixels. For quantification, a fixed ROI (Region of Interest) of 35 μm × 25 μm was generated and saved as a template in ZEN software (Response Figure-2). Because all images were acquired under identical imaging parameters, the same ROI template was applied uniformly across all images.

To ensure that the ROI was positioned in the same anatomical region in every animal, all images used for lysosome quantification and cuticle morphology analysis were acquired from a defined body region located posterior to the head (terminal pharyngeal bulb) and anterior to the vulva (Response Figure). The ROI was placed within this region using these anatomical landmarks as references and applied consistently across all worms analyzed. This procedure is now described in detail in the Methods section- "Quantification of lysosomal morphology".

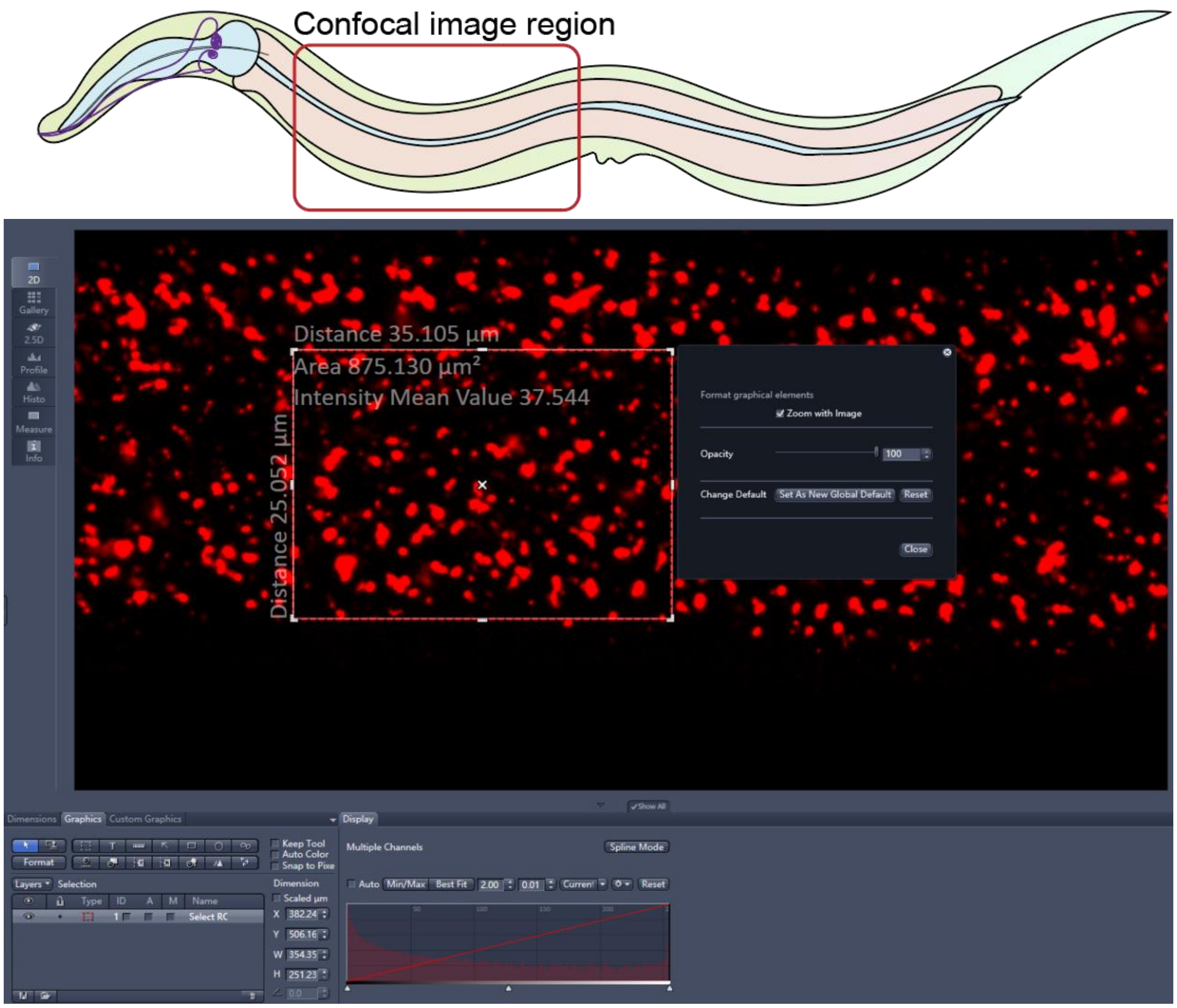

Response Figure-2: The image illustrates how we select an ROI (Region of Interest) for lysosomal observation in animals.

2G and 2J: Because heat shock is used to induce the expression of the NUC-1 reporters, more than one biological replicate should be performed.

Response:

We confirm that all heat-shock experiments were performed with at least three independent biological replicates (each comprising $n \geq 18–20$ animals per condition). To make this explicit, we have updated the figure legends for Fig. 2G, 2J, and all related panels to clearly state the number of biological replicates and the total number of animals analyzed.

2G, 2J, and 2M: Why are dots for values from individual worms not plotted as they are in 2B,2D,

and 2E?

Response:

Thank you for pointing this out. We have updated Fig. 2G, 2J, and 2M (and other equivalent panels across the manuscript) to overlay individual-worm data points on the bar/box plots.

3C: Why were L1s used for the proteomics when all other experiments were performed on animals 24 hours post L4?

Response:

We chose synchronized L1-stage animals for the secretome screen for two specific reasons that we have now added to the manuscript.

First, bleach-synchronized L1 animals have not yet been exposed to any bacterial food source and therefore have no prior innate-immune "history." When these naïve L1s are then exposed to PA14, the secreted proteins they produce should reflect the direct response to PA14 itself, without confounding by adaptive responses to a preceding E. coli OP50 diet. In contrast, L4 animals have been on E. coli for ~48 h and may already exhibit some baseline innate-immune adaptation that could blunt or alter their response upon PA14 transfer.

Second, our own silver-staining results (Response Figure-3) showed that the differences in secreted-protein profiles between OP50- and PA14-exposed worms were substantially more pronounced in L1s than in L4s, giving L1s greater discovery power for PA14-specific factors.

Once NSIF-1 was identified, we used L4+24 h young adults for all downstream phenotypic, genetic, and functional assays because cuticle, lysosomal, and survival phenotypes are most readily and reproducibly scored at this stage. This rationale has been added to the Methods section- “Isolation of secreted proteins from *C. elegans* ”.

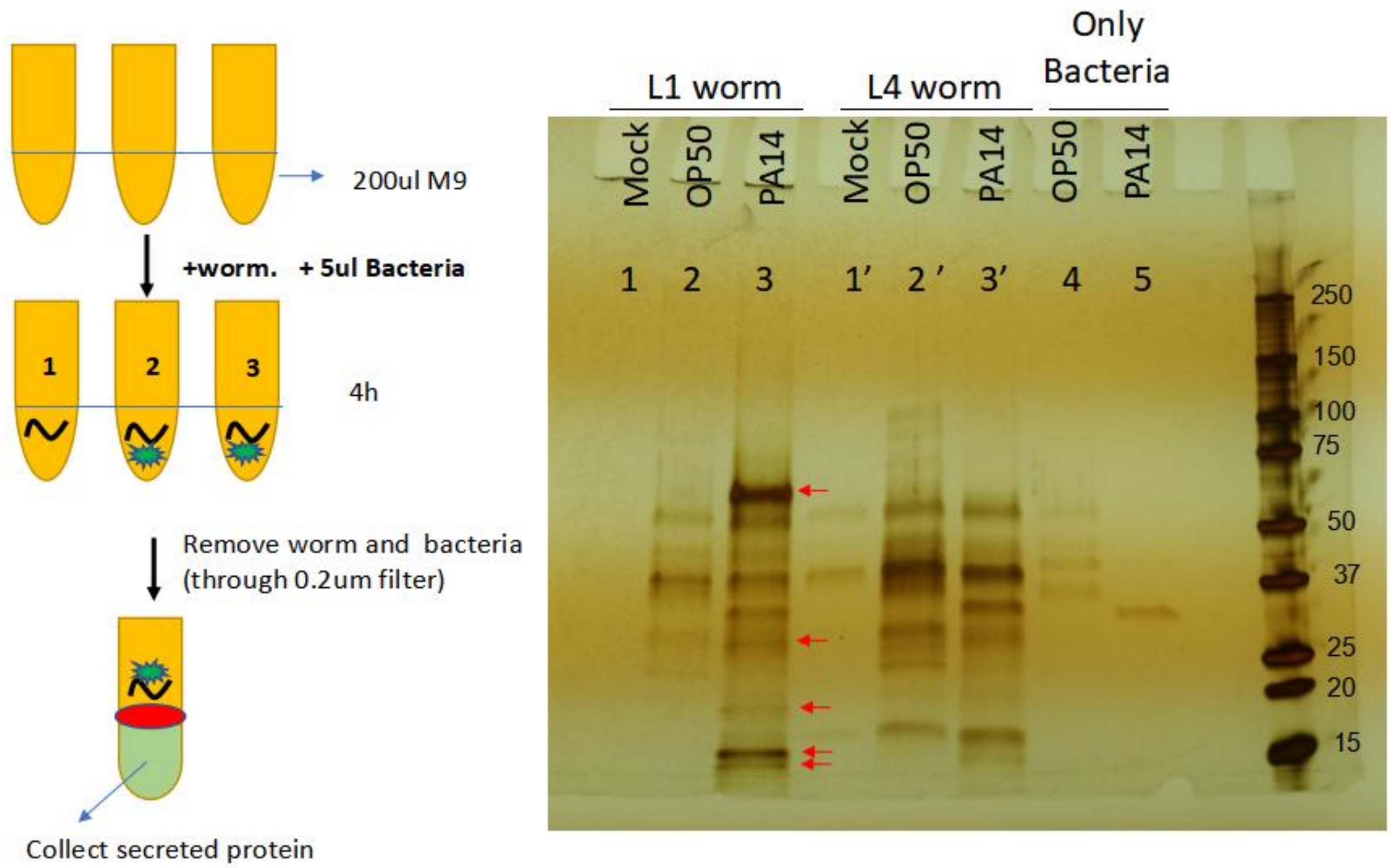

Response Figure-3.

Silver staining of secreted proteins from wild-type (WT) animals under different treatment conditions. L1-stage or L4-stage animals were grown in liquid culture for 4 hours (lane 1, 1') or exposed to *E. coli* OP50 (lane 2, 2') or *P. aeruginosa* PA14 (lane 3, 3'). Control lanes 4 and 5 contained the same liquid culture treated with OP50 or PA14, respectively, but without animals. After treatment, animals and bacteria were removed by centrifugation, and the medium was filtered through a 0.22μm filter to obtain secreted proteins for sliver staining analysis.

3C: How is this data normalized?

Response:

Each lysosome's fluorescence intensity was divided by the mean fluorescence intensity of all control-group lysosomes. Specifically, for the control group, each lysosome's value was normalized to the control mean (yielding a distribution centered around 1.0). For the mutant group, each lysosome's value was likewise divided by the control mean, so that the mutant values can be read as fold-change relative to control. This rationale has been added to the Methods section- "Quantification of NUC-1::pHTomato fluorescence intensity ".

2F: Is this data the same as one of the plots in S7 with the overexpression strain removed? If not,

where are the additional biological replicates for the survival of WT and nsif-1 mutant after infection?

Response:

We apologize for the confusion. The survival data in Fig. 3F (WT vs *nsif-1*) and the WT/*nsif-1* traces shown in Appendix Fig. S7 (Now in S8 Fig) are independent biological replicates, not the same dataset re-plotted. All PA14 survival assays in the manuscript were performed in three independent biological replicates. We have added a clarifying note in the figure legends

S7: What promoter is used for the expression of the full-length nsif-1 here? Are these transgenic animals expressing extrachromosomal arrays, or is this integrated?

Response:

The full-length NSIF-1 used in Appendix Fig. S7 (now in S8 Fig) is driven by its own native promoter (Pnsif-1). The strain used is the integrated transgenic line YNU22: *unc-76(e911);ylfIs1*[P_{nsif-1}NSIF-1::GFP*; unc-76*(+)], not an extrachromosomal array. This information has been added to the figure legend.

S7: Does overexpression of nsif-1 cause any changes to the cuticle structure or the hypodermal lysosomes?

Response:

Because loss of nsif-1 enhances lysosomal function and produces a denser cuticle, NSIF-1 acts as a negative regulator, and overexpression could in principle perturb these phenotypes. We tested this directly. Overexpression of either full-length NSIF-1 or ΔSP NSIF-1 under the native P*nsif-1* promoter in wild-type animals produced no detectable change in hypodermal lysosomal morphology or cuticle organization relative to wild type under OP50 feeding condition. In contrast, when the same constructs were driven by the hypodermal *col-12* promoter, both full-length NSIF-1 and ΔSP NSIF-1 markedly disrupted lysosomal morphology and cuticle organization, phenocopying PA14 infection (New data: S20 Fig).

These results indicate two things: (i) native-level overexpression of NSIF-1 in its normal expressing cells is well tolerated, consistent with our finding that PA14-induced upregulation, not basal expression, drives pathology; and (ii) forced expression of NSIF-1 directly in the hypodermis is sufficient to recapitulate the lysosomal and cuticle defects, supporting the conclusion that the hypodermis is the site of action. We added this at Results section - "NSIF-1 impairs lysosomal function via inhibiting ELT-3"

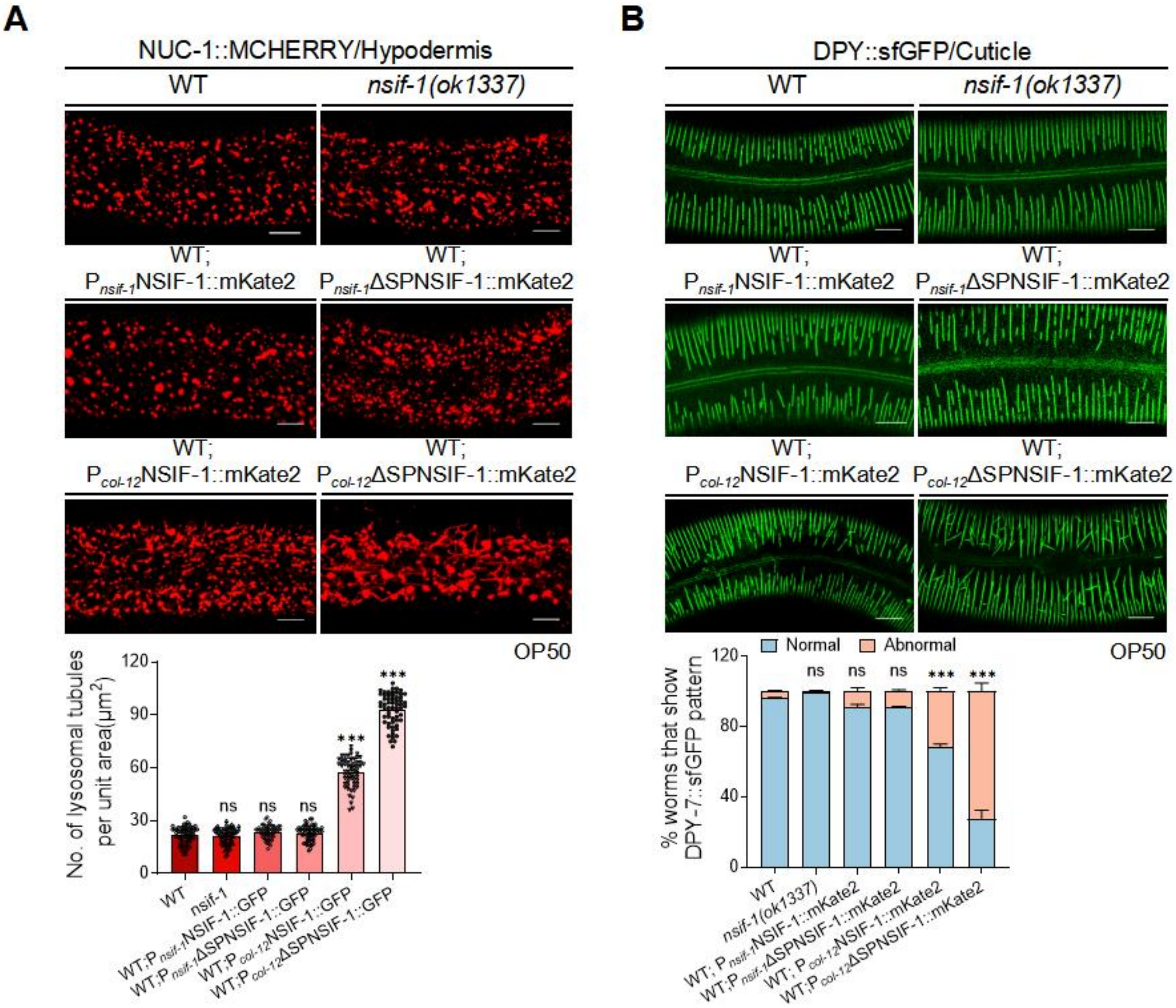

S20 Fig. Epidermal expression of NSIF-1 is sufficient to impact lysosomal homeostasis and cuticle integrity.

(A) Confocal fluorescence imaging and quantification of NUC-1::mCherry-labeled lysosomes in wild-type (WT), *nsif-1(ok1337)* mutants, and WT animals expressing NSIF-1::mKate2 or the signal-peptide-deleted variant (ΔSP-NSIF-1::mKate2) under the control of the endogenous *nsif-1* promoter (P*nsif-1*) or the epidermal promoter (P*col-12*). Quantification was performed on 60 animals (n = 60).

Scale bars: 10 μm.

(B) Confocal fluorescence imaging and quantification of DPY-7::sfGFP-marked cuticle in WT, *nsif-1(ok1337)* mutants, and WT animals expressing NSIF-1::mKate2 or the signal-peptide-deleted variant (ΔSP-NSIF-1::mKate2) under the control of the endogenous *nsif-1* promoter (P*nsif-1*) or the epidermal promoter (P*col-12*). Quantification was performed on 300 animals (n = 300). Scale bars: 10 μm.

For all quantification, data are presented as mean ± SEM. Statistical comparisons were performed using one-way ANOVA followed by Tukey's multiple comparisons test. ***$p<0.001$, ns., not significant. All experiments were performed independently at least three times. The data underlying this figure can be found in S2 Data.

2G and S8: Colocalization of the nsif-1 translational reporter with a neuronal-specific reporter should be performed, and images of a whole animal should be shown. This is particularly relevant because rgef-1 is used for neuronal expression later in the paper, but from the images presented, it is not clear how widespread nsif-1 is expressed in neurons.

Response:

We thank the reviewer for this valuable suggestion. To determine the expression pattern, we performed colocalization of the P*nsif-1::*GFP and P*nsif-1*::NSIF-1::GFP reporters with well-characterized neuronal markers, including those for ASI and ASJ neurons at the whole worm level (New data: S14 Fig, and Fig 4D-E). These new images confirm that *nsif-1* is predominantly expressed in ASI and ASJ sensory neurons under our imaging conditions.

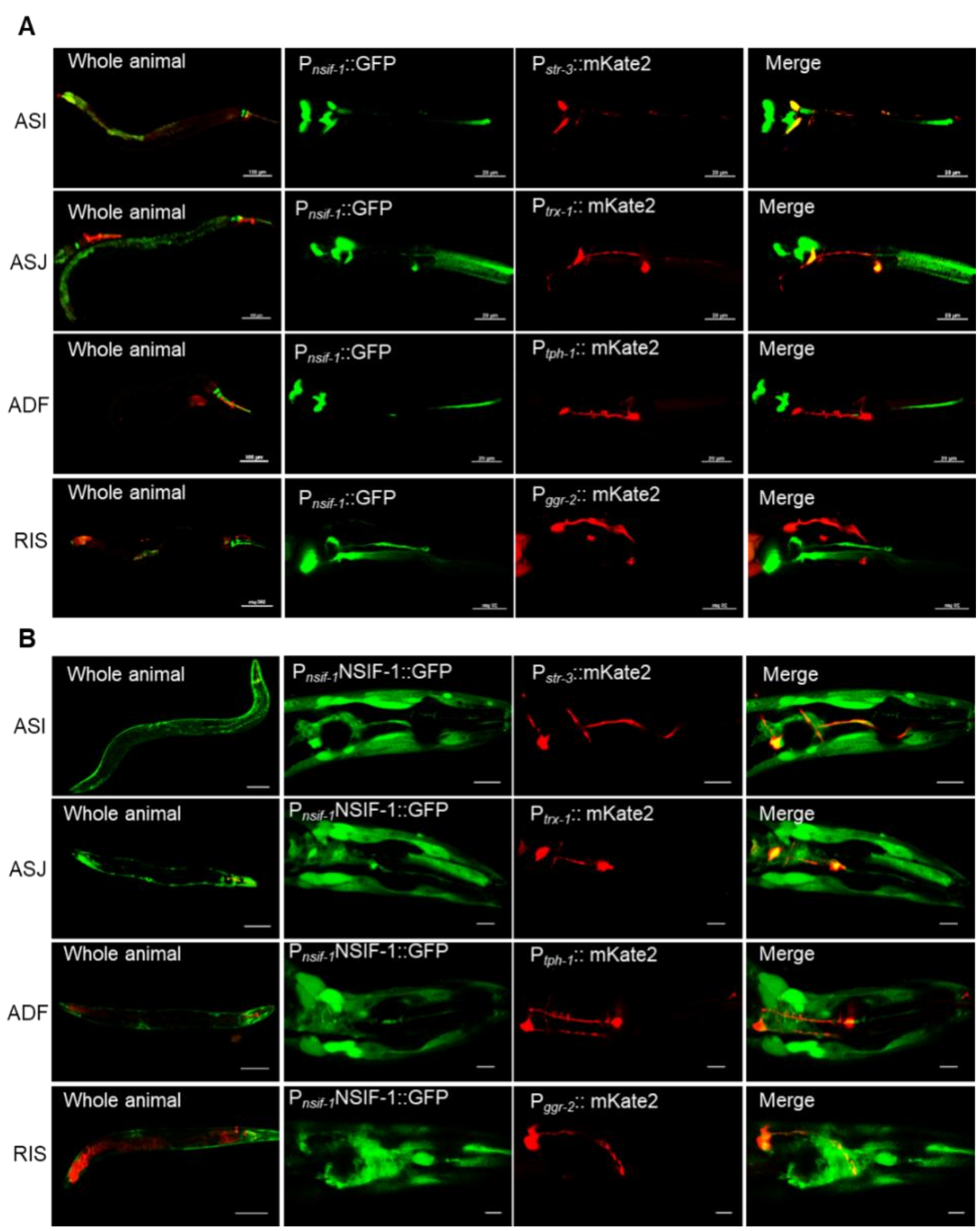

S14 Fig. Co-localization analysis of NSIF-1 transcriptional and translational reporters with ASI, ASJ, ADF, and RIS neurons.
(A) Localization of the NSIF-1 transcriptional reporter ($P_{nsif\text{-}1}$::GFP) in ASI ($P_{str\text{-}3}$::mKate2), ASJ ($P_{trx\text{-}1}$::mKate2), ADF ($P_{tph\text{-}1}$::mKate2), and RIS ($P_{ggr\text{-}2}$::mKate2) neurons. Scale bars: whole animal, 100 µm; others, 20 µm.
(B) Localization of the NSIF-1 translational reporter ($P_{nsif\text{-}1}$NSIF-1::GFP) in ASI ($P_{str\text{-}3}$::mKate2), ASJ ($P_{trx\text{-}1}$::mKate2), ADF ($P_{tph\text{-}1}$::mKate2), and RIS ($P_{ggr\text{-}2}$::mKate2) neurons. Scale bars: whole animal, 100 µm; others, 10 µm.

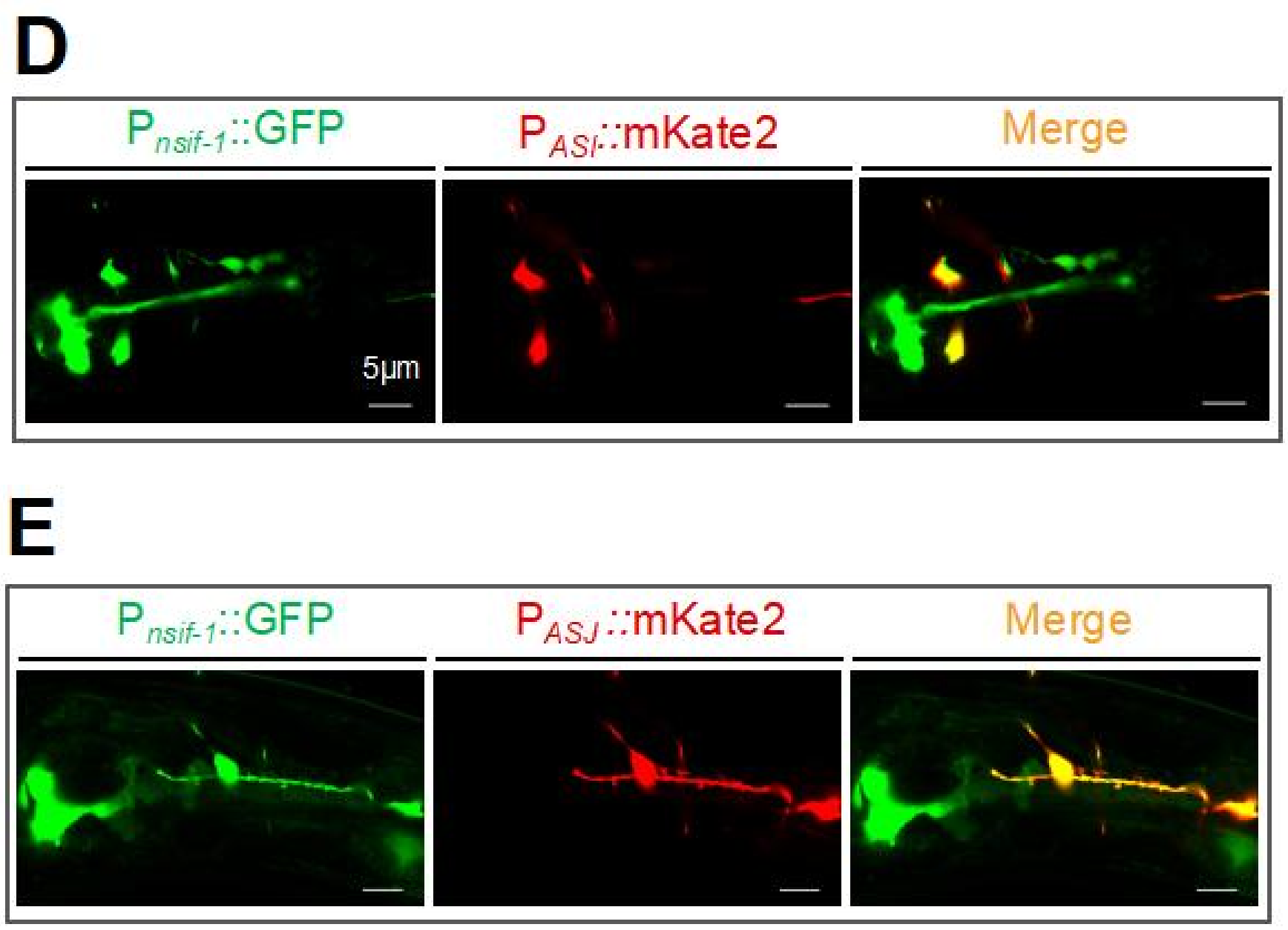

Fig 4 (D-E) Analysis of the localization distribution of $P_{nsif\text{-}1}$::GFP in ASI($P_{ASI\ (str\text{-}3)}$::mKate2) (D) and ASJ ($P_{ASJ(trx\text{-}1)}$::mKate2) (E) neurons for adult animals. Scale bars, 5µm.

From the pictures presented, it is not possible to tell if the nsif-1 translational reporter is not expressed in the hypodermis. Again, colocalization with a hypodermal reporter and whole worm images would make this clear.

Response:

To directly visualize any hypodermal expression, we crossed the endogenous CRISPR knock-in strain *syb9508* (NSIF-1::mNeonGreen::3xFLAG) with a hypodermal marker (P*col-12*::mKate2) and acquired whole-animal images. We observe robust mNeonGreen signal in the hypodermis that overlaps with the Pcol-12::mKate2 marker (New data: S9B Fig).

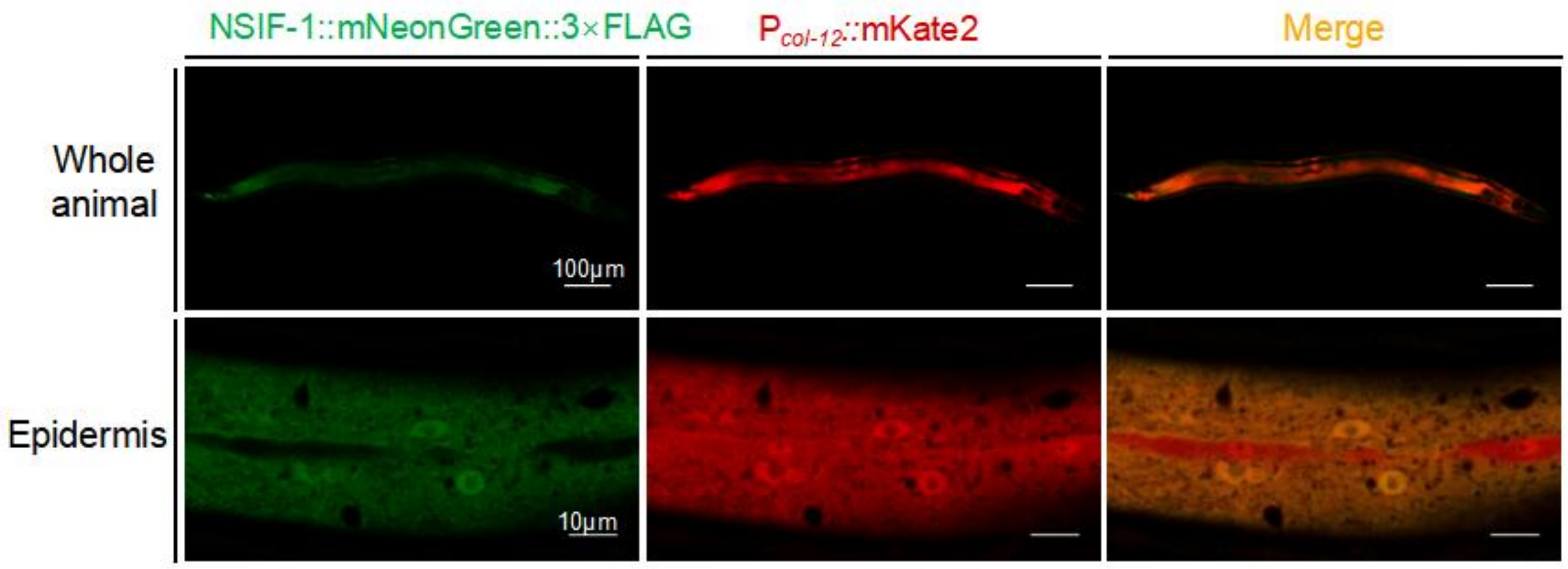

S9 Fig (B) Confocal fluorescence imaging of the CRISPR knock-in strain NSIF-1::mNeonGreen::3×FLAG (*syb9508*), showing that the tagged protein colocalizes with the epidermis, which is marked by P*col-12*::mKate2. Scale bars: 100 µm (in whole animals) and 10 µm (in epidermis).

EV4A and 4B: What p-value and fold change cut-off were used to determine differentially expressed genes in the nsif-1 mutant

Response:

We thank the reviewer for this comment. EV4A and 4B have been renumbered as S15A and S15B in the supplementary figures. Differentially expressed genes (DEGs) were defined as those with a false discovery rate (FDR) < 0.05 and a fold change ≥ 1.5 . We have now clarified these criteria in the corresponding figure legends.We have now clarified these criteria in the figure legends of S15A-S15B Fig.

EV4C: No statistics are presented here or for the RNA seq, so it is not appropriate to say these genes are "significantly upregulated".

Response:

We have now performed qRT-PCR to validate the RNA-seq data (New data: S15C Fig). For each gene, three biological replicates, each with three technical replicates, were analyzed. Statistical significance was determined using Student's test.

S17: Why were L1s used for the RNA-seq when all other experiments were performed on animals

24 hours post L4?

Response:

The reasoning is the same as for the proteomics (see our response for: [3C: Why were L1s used for the proteomics when all other experiments were performed on animals 24 hours post L4?]).

Synchronized L1 animals are naïve to bacterial food and therefore allow detection of transcriptional changes attributable to *nsif-1* loss without prior bacterial-immune-history confounds. We chose this stage specifically to enrich for direct, intrinsic *nsif-1*-regulated transcriptional changes. Phenotypic and survival assays were then conducted on L4 + 24 h young adults because these are the stages at which cuticle, lysosomal, and host-defense phenotypes are most robustly and reproducibly assayed.

EV5A and 5B: Does hypodermal expression of NSIF-1::GFP rescue the mutant phenotypes?

Response:

Yes -this point is addressed in our response to above question ("Demonstrate that NSIF-1 expressed in the hypodermis rescues the nsif-1 mutant phenotypes when infected with PA14."). Briefly, expression of full-length NSIF-1::GFP and ΔSP NSIF-1::GFP under the hypodermal *col-12* promoter in the *nsif-1(ok1337)* background reverses the protective lysosomal and cuticle phenotypes of the mutant under PA14 infection (New data: S16C-D Fig), demonstrating that the hypodermis is the relevant site of NSIF-1 action.

The rationale for removing the SP from hypodermally expressed NSIF-1:GFP is unclear. Just because elt-3-regulated genes are deregulated in nsif-1 mutants does not mean that NSIF-1 must function in the nucleus. **Does NSIF-1 have an NLS? How could it get diverted from the secretory pathways to the nucleus?**

Response:

We thank the reviewer for raising this important mechanistic question. Below we address the three specific points raised: (1) the rationale for removing the signal peptide (SP) from hypodermally expressed NSIF-1, (2) whether NSIF-1 contains a nuclear localization signal (NLS), and (3) how NSIF-1 could potentially be diverted from the secretory pathway to the nucleus.

1. Rationale for removing the signal peptide from hypodermally expressed NSIF-1::GFP

The rationale is rooted in the fundamental biology of protein secretion. For classical secreted proteins, the signal peptide (SP) is cleaved by signal peptidase in the endoplasmic reticulum (ER) lumen immediately after co-translational translocation. The SP is removed, and the mature protein is released into the ER lumen, subsequently trafficked through the Golgi and secreted to the

extracellular space. Therefore, if NSIF-1 is secreted from neurons to the hypodermis, the form that reaches the hypodermal cells should be the mature, SP-cleaved version.

To test whether this mature form can directly act in the hypodermis to regulate lysosomal function and cuticle integrity, we expressed a signal-peptide-deleted (ΔSP) NSIF-1 specifically in the hypodermis under the *col-12* promoter. Our results showed that hypodermal overexpression of ΔSP NSIF-1 markedly disrupted lysosomal morphology and cuticle organization, phenocopying the effects of PA14 infection (**S20A-B Fig**). This demonstrates that the mature, SP-cleaved form of NSIF-1 is sufficient to exert its inhibitory effects on hypodermal lysosomal function. Thus, the ΔSP construct was designed to mimic the physiologically relevant mature form of NSIF-1 that is secreted from neurons and taken up by hypodermal cells.

2. Does NSIF-1 contain an NLS?

Yes. Using cNLS Mapper (https://nls-mapper.iab.keio.ac.jp/), we identified a putative NLS sequence within NSIF-1 with a score of 3.3 (S21 Fig). According to the cNLS Mapper scoring system established by Kosugi et al. (2009), scores of 3-5 indicate proteins that localize to both the nucleus and the cytoplasm. This prediction is consistent with our observation that ΔSP NSIF-1 (which lacks the SP and therefore cannot enter the ER–Golgi secretory pathway) accumulates in the nucleus (**S22A Fig**). The NLS is located downstream of the SP, near the middle of the protein, which is consistent with the architecture of other proteins that have both secretory and nuclear targeting signals.

3. How could NSIF-1 be diverted from the secretory pathway to the nucleus?

While we do not yet know the precise molecular mechanism by which NSIF-1 is diverted from the secretory pathway to the nucleus, there are well-established precedents for such a phenomenon in other proteins (see below example 1 and 2) that possess both an N-terminal SP and an internal NLS.

Example 1: Parathyroid hormone-related protein (PTHrP) is a classic example. PTHrP contains both an N-terminal ER-targeting signal peptide and an internal NLS. Studies have demonstrated that the cleavage efficiency of PTHrP's signal peptide is cell-type-specific. There is an inverse relationship between signal peptide cleavage and nuclear localization: when the SP is inefficiently cleaved, the protein can be diverted from the secretory pathway and instead enter the nucleus (Amaya Y et al, 2008) .

Example 2: Fibroblast growth factor 3 (FGF3) is yet another example where competition between nuclear localization and secretory signal within the same protein determines its subcellular fate (Kiefer, P et al, 1994).

While the precise molecular mechanism by which NSIF-1 is diverted from the secretory pathway to the nucleus remains to be determined, the nuclear targeting of secretory proteins such as PTHrP and FGF represents an unconventional mode of signaling that complements classical receptor-mediated pathways. These proteins can access the nucleus through multiple mechanisms (Henderson JE 1997), any of which could potentially apply to NSIF-1: (1) receptor-mediated endocytosis followed by retrograde trafficking to the nucleus, often in association with their receptors or other carriers; (2) alternative translation initiation that deletes the signal peptide and exposes an NLS, generating isoforms that bypass the secretory pathway; (3) regulation of signal peptide cleavage efficiency that determines the balance between secretion and nuclear entry—as demonstrated for PTHrP, where inefficient cleavage due to a less hydrophobic SP allows a fraction of the protein to escape ER targeting and be redirected to the nucleus; and (4) interaction with specific nuclear transport receptors such as importins, which recognize the internal NLS and mediate nuclear import.

In our model, we now propose that neuronal NSIF-1 is secreted and subsequently taken up by hypodermal cells. Because the signal peptide is co-translationally removed during biogenesis in the neuron, the internalized protein in the hypodermal cell is already the mature (SP-cleaved) form, yet retains its intact internal NLS. Once inside the hypodermal cell, this mature protein may escape from endosomal compartments into the cytosol and then translocate to the nucleus via NLS-mediated import. Consistent with this model, our ΔSP NSIF-1 construct—which lacks the SP and therefore cannot enter the ER-Golgi secretory pathway—accumulates efficiently in the nucleus when expressed in the hypodermis (S22A Fig), thereby functionally recapitulating the proposed nuclear entry of the mature, internalized form.

Acknowledgement of limitations

We acknowledge that the precise mechanism by which internalized NSIF-1 escapes the endosomal compartment and translocates to the nucleus remains unknown. Identifying the receptor(s) mediating NSIF-1 uptake and the endosomal escape machinery will be important directions for future studies. Additionally, whether SP cleavage efficiency or alternative trafficking routes contribute to NSIF-1 nuclear localization in vivo during infection requires further investigation. We have noted these limitations in the revised manuscript (Discussion/Limitations section). Our data collectively provide a coherent mechanistic framework, but we recognize that detailed dissection of the nuclear entry pathway is beyond the scope of the current study.

Reference:

Amaya Y, Nakai T, Komaru K, Tsuneki M, Miura S. Cleavage of the ER-targeting signal sequence

of parathyroid hormone-related protein is cell-type-specific and regulated in Cis by its nuclear localization signal. J Biochem. 2008;143:569–579.

Kiefer, P., Acland, P., Pappin, D. et al. Competition between nuclear localization and secretory signals determines the subcellular fate of a single CUG-initiated form of FGF3.. EMBO J 13, 4126–4136 (1994). https://doi.org/10.1002/j.1460-2075.1994.tb06730.x

Henderson JE. Nuclear targeting of secretory proteins. Mol Cell Endocrinol. 1997 Apr 25;129(1):1-5. doi: 10.1016/s0303-7207(97)04021-5. PMID: 9175623.

Kosugi S., Hasebe M., Tomita M., and Yanagawa H. (2009) Systematic identification of yeast cell cycle-dependent nucleocytoplasmic shuttling proteins by prediction of composite motifs. Proc. Natl. Acad. Sci. USA 106, 10171-10176

5B and 5C: How was effective knockdown of nsif-1 verified?

Response:

To verify the efficacy of tissue-specific RNAi of *nsif-1* in ASI/ASJ neurons, we used the *qxIs383; sid-1(qt9)* systemic-RNAi-null background and restored *sid-1* specifically in ASI or ASJ neurons by expressing SID-1::3×FLAG under the *str-3* (ASI) or *trx-1* (ASJ) promoter, together with the corresponding ASI/ASJ-driven NSIF-1::GFP reporter. Comparing animals fed *nsif-1* RNAi versus the HT115 empty-vector control, NSIF-1::GFP fluorescence in ASI/ASJ was markedly and significantly reduced upon *nsif-1* RNAi, confirming efficient neuron-specific knockdown (New data: Figure 6A-B). As a functional readout, the lysosomal morphology under PA14 infection in the *nsif-1* RNAi animals matched that of *nsif-1(ok1337)* loss-of-function (reduced tubular lysosomes), further validating the system.

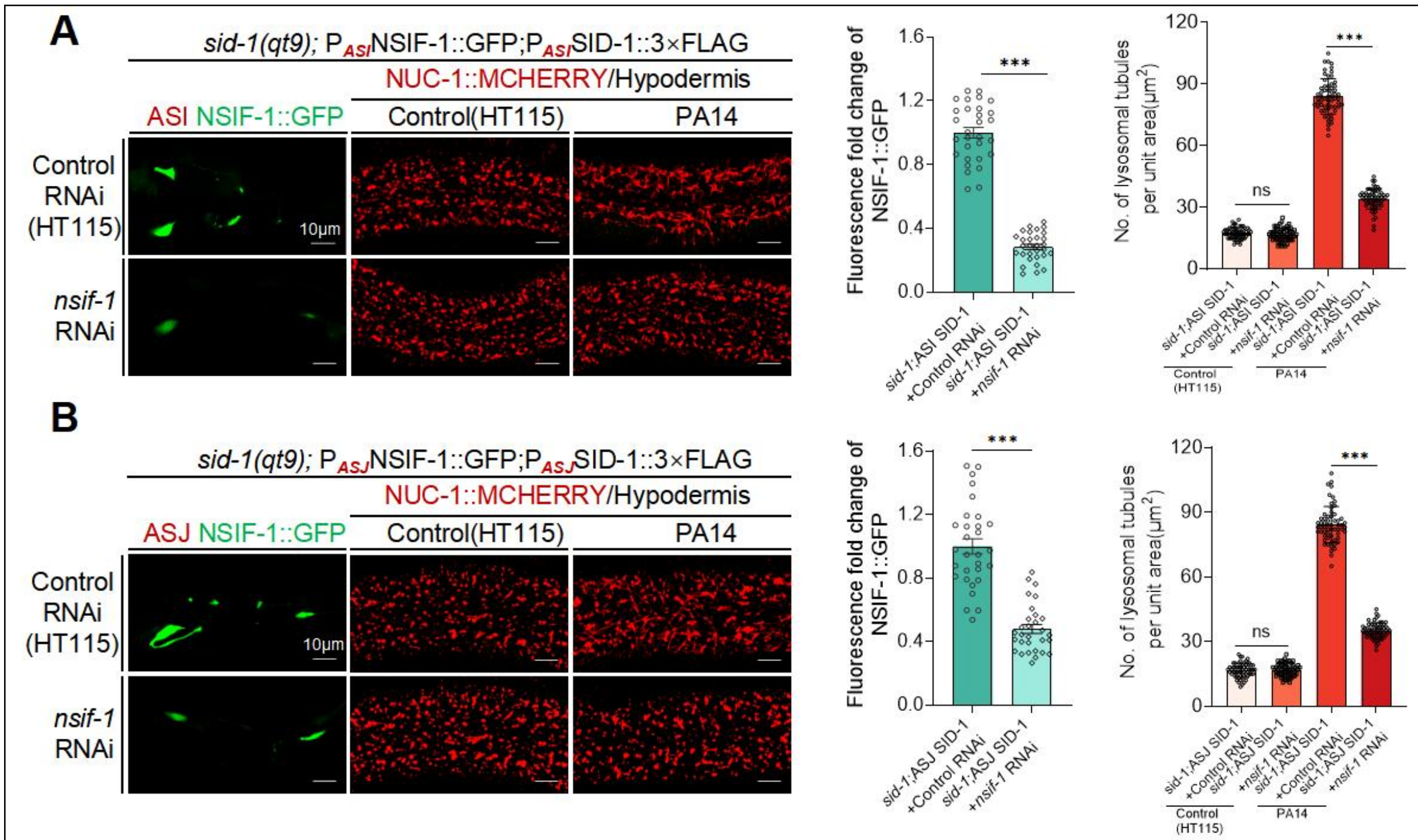

Figure 6

(A) Confocal microscopy images and quantification of ASI neuron-specific *nsif-1* knockdown efficiency and lysosomal morphology. Compared with Control RNAi, ASI neuron-specific *nsif-1* knockdown significantly reduced NSIF-1::GFP fluorescence intensity in ASI neurons and decreased the number of tubular lysosomes following 24-h PA14 exposure (infection initiated at the L4 stage). NSIF-1::GFP fluorescence intensity was quantified in 30 worms. Tubular lysosomes were quantified by counting three 35×25μm² regions per worm (n=60 worms). Scale bars, 10μm.

(B) Confocal microscopy images and quantification of ASJ neuron-specific *nsif-1* knockdown efficiency and lysosomal morphology. Compared with Control RNAi, ASI neuron-specific *nsif-1* knockdown significantly reduced NSIF-1::GFP fluorescence intensity in ASI neurons and decreased the number of tubular lysosomes following 24-h PA14 exposure (infection initiated at the L4 stage). NSIF-1::GFP fluorescence intensity was quantified in 30 worms. Tubular lysosomes were quantified by counting three 35×25μm² regions per worm (n=60 worms). Scale bars, 10μm.

S14 and S15: How was the efficacy of ASI/ASJ ablation confirmed?

Response:

To confirm efficacy of EGL-1-mediated ASI/ASJ ablation, we co-expressed cell-death activator EGL-1 (under the *str-3* or *trx-1* promoter) with ASI/ASJ-specific NSIF-1::GFP in the *qxIs383* background. Compared with sibling controls lacking EGL-1, animals carrying the EGL-1 transgene showed a marked and significant reduction in NSIF-1::GFP fluorescence in the targeted neurons, consistent with successful cell ablation (New data: S18A Fig). The functional readout — lysosomal morphology under PA14 infection — also phenocopied the *nsif-1* RNAi/loss-of-

function condition (Fig 5A-B, S18A), confirming the biological efficacy of the ablation.

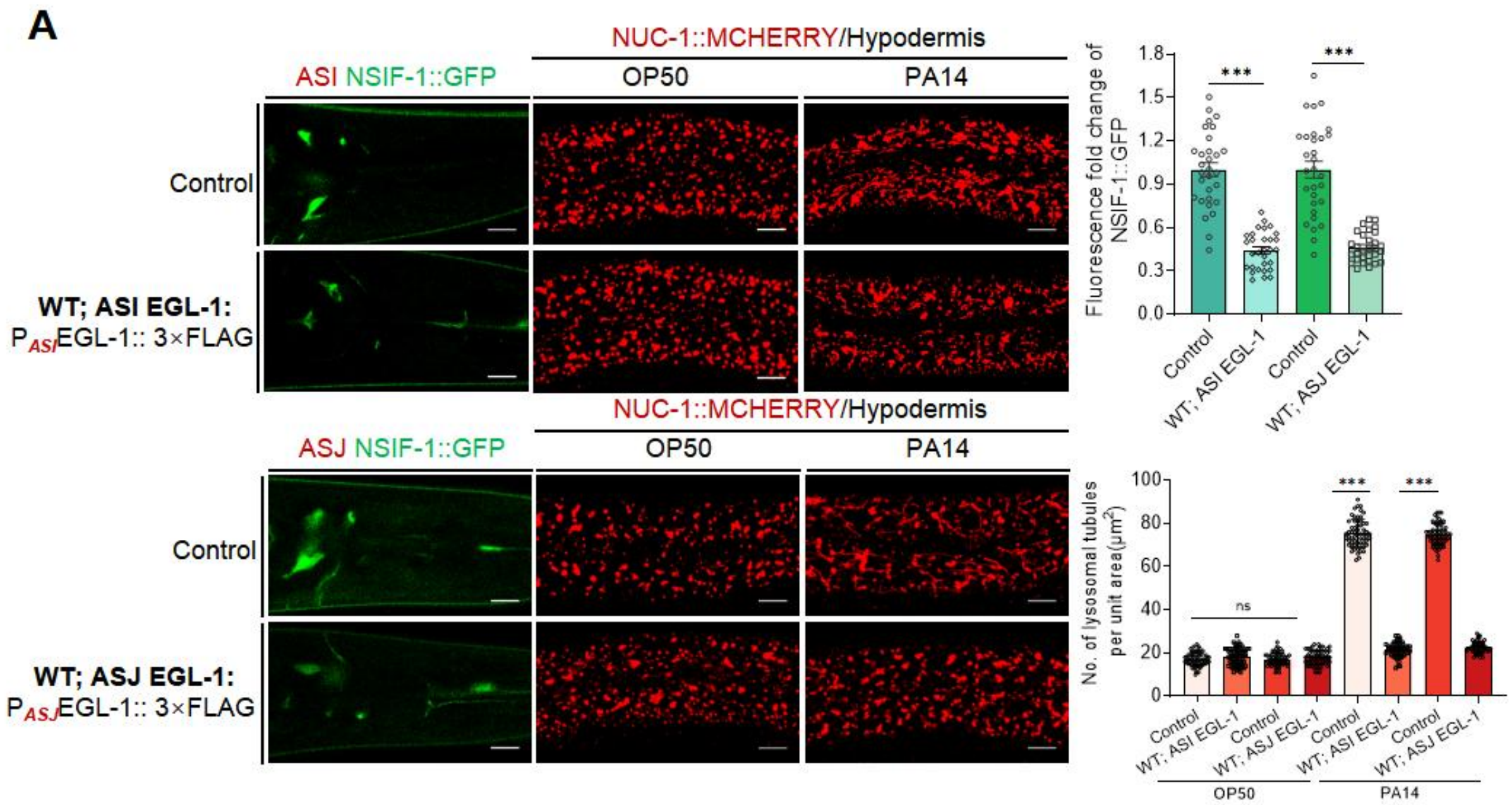

S18 Fig. (A) Confocal microscopy images and quantification of ASI/ASJ neuron ablation efficiency and epidermal lysosomal morphology. EGL-1-mediated genetic ablation of ASI and ASJ neurons resulted in a significant reduction of NSIF-1::GFP fluorescence in these neurons. Following 24 h of PA14 exposure (initiated at the L4 larval stage), ablation of ASI/ASJ neurons prevented the PA14-induced reduction in tubular lysosomes. NSIF-1::GFP fluorescence intensity was quantified in 30 worms. Tubular lysosomes were quantified by counting three 35 × 25 μm² regions per worm (n=60 worms).

6A: From the images, it looks like ELT-3:GFP is more highly expressed in the nsif-1 mutant, but not that the localization has changed. This can be simply tested by qRT-PCR to quantify the level of endogenously expressed elt-3 mRNA.

Response:

We performed the requested qRT-PCR on synchronized L4 + 24 h wild-type and *nsif-1(ok1337)* animals (three biological replicates, three technical replicates per biological replicate). We find that endogenous *elt-3* mRNA is significantly upregulated in *nsif-1(ok1337)* compared with wild type (New data: S22C Fig). We have therefore revised the text to indicate that NSIF-1 loss leads to both increased *elt-3* expression and increased nuclear ELT-3::GFP signal, rather than describing the change as a localization shift alone.

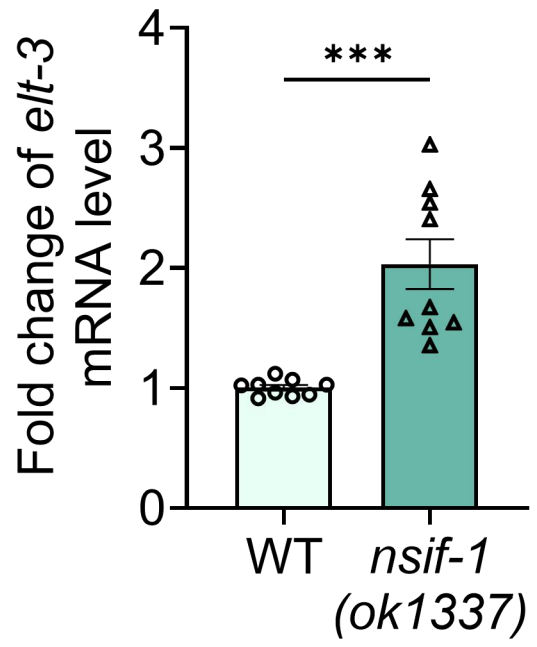

S22 Fig (C) qRT-PCR analysis of *elt-3* expression changes in adult (L4+24h) WT and *nsif-1(ok1337)* animals. Three biological replicates, each with three technical replicates, were analyzed for *elt-3*.
