# Supplementary material for: Pathogen subversion of neuro-epidermal signaling impairs lysosomal function to disrupt collagen homeostasis in Caenorhabditis elegans
Source: PLoS Biol. 2026 Sep 22;24(9):e3004016. doi: 10.1371/journal.pbio.3004016 (PMC13649122; doi:10.1371/journal.pbio.3004016)
Supplement: Peer review history

# Editor report: Decision Letter 0

**DOI:** 10.1371/journal.pbio.3004016.r001

**Authors:** Ines Alvarez-Garcia

16 Mar 2026

Dear Dr, Qi

Thank you for submitting the revision of your manuscript entitled "Pathogen Subversion of Neuro-Epidermal Signaling Impairs Lysosomal Function to Disrupt Collagen Homeostasis" for consideration as a Research Article by PLOS Biology. Please also accept my apologies for the delay in contacting you with a decision - it took a while to obtain all the advice and information needed in order for us to proceed.

Your manuscript has now been evaluated by the PLOS Biology editorial staff as well as by an academic editor with relevant expertise and I am writing to let you know that we would like to send your submission out for external peer review.

However, before we can send your manuscript to reviewers, we need you to complete your submission by providing the metadata that is required for full assessment. To this end, please login to Editorial Manager where you will find the paper in the 'Submissions Needing Revisions' folder on your homepage. Please click 'Revise Submission' from the Action Links and complete all additional questions in the submission questionnaire.

Once your full submission is complete, your paper will undergo a series of checks in preparation for peer review. After your manuscript has passed the checks it will be sent out for review. To provide the metadata for your submission, please Login to Editorial Manager (https://www.editorialmanager.com/pbiology) within two working days, i.e. by Mar 18 2026 11:59PM.

Feel free to email us at plosbiology@plos.org if you have any queries relating to your submission.

Kind regards,

Ines

--

Ines Alvarez-Garcia, PhD

Senior Editor

PLOS Biology

ialvarez-garcia@plos.org

# Peer review: Decision Letter 1

**DOI:** 10.1371/journal.pbio.3004016.r002

**Authors:** Ines Alvarez-Garcia

20 Apr 2026

Dear Dr Qi,

Thank you for your patience while we considered the revised version of your manuscript entitled "Pathogen Subversion of Neuro-Epidermal Signaling Impairs Lysosomal Function to Disrupt Collagen Homeostasis" for publication as a Research Article at PLOS Biology. Your revised study has been evaluated by the PLOS Biology editors, an Academic Editor with suitable expertise, two of the original reviewers of the previous version and a new reviewer.

The reviews are attached below. As you will see, while Reviewers 1 and 2 are mostly positive, Reviewer 2 agrees with the prior concerns raised by the original Reviewer 3 about the staging of the timecourse and potential molting artefacts that could explain the results. Thus, Reviewer 2 thinks that you should perform an experiment where dpy-7::GFP young adults or L4+1 day animals are analysed, placed on PA14 and imaged 0, 3, 6, 12, and 24 hours later to address this issue. In addition, Reviewer 4 raises concerns with the lack of attention to detail throughout and misses important reporting and methodological details. This reviewer also notes that the evidence that NSIF-1 secreted from ASI/ASJ and functions in the hypodermis presented in the paper is not complete, and provides several experimental requests to provide more direct evidence for the model.

In light of the reviews and after consulting with the Academic Editor, we would like to invite you to revise the work to thoroughly address the reviewers' reports. Given the extent of revision needed, we cannot make a decision about publication until we have seen the revised manuscript and your response to the reviewers' comments. Your revised manuscript is likely to be sent for further evaluation by all or a subset of the reviewers.

In addition to these revisions, you will need to complete some formatting changes, which you will receive in a follow up email. A member of our team will be in touch with a set of requests shortly.

We expect to receive your revised manuscript within 3 months. Please email us (plosbiology@plos.org) if you have any questions or concerns, or would like to request an extension.

At this stage, your manuscript remains formally under active consideration at our journal; please notify us by email if you do not intend to submit a revision so that we may withdraw it.

**IMPORTANT - SUBMITTING YOUR REVISION**

Your revisions should address the specific points made by each reviewer. Please submit the following files along with your revised manuscript:

1. A 'Response to Reviewers' file - this should detail your responses to the editorial requests, present a point-by-point response to all of the reviewers' comments, and indicate the changes made to the manuscript.

*NOTE: In your point-by-point response to the reviewers, please provide the full context of each review. Do not selectively quote paragraphs or sentences to reply to. The entire set of reviewer comments should be present in full and each specific point should be responded to individually, point by point.

You should also cite any additional relevant literature that has been published since the original submission and mention any additional citations in your response.

2. In addition to a clean copy of the manuscript, please also upload a 'track-changes' version of your manuscript that specifies the edits made. This should be uploaded as a "Revised Article with Changes Highlighted" file type.

3. Resubmission Checklist

When you are ready to resubmit your revised manuscript, please refer to this resubmission checklist: https://plos.io/Biology_Checklist

To submit a revised version of your manuscript, please go to https://www.editorialmanager.com/pbiology/ and log in as an Author. Click the link labelled 'Submissions Needing Revision' where you will find your submission record.

Please make sure to read the following important policies and guidelines while preparing your revision and fulfil the editorial requests:

a) *PLOS Data Policy*

Please note that as a condition of publication PLOS' data policy (http://journals.plos.org/plosbiology/s/data-availability) requires that you make available all data used to draw the conclusions arrived at in your manuscript. If you have not already done so, you must include any data used in your manuscript either in appropriate repositories, within the body of the manuscript, or as supporting information (N.B. this includes any numerical values that were used to generate graphs, histograms etc.). Please also indicate in each figure legend where the data can be found. For an example see here: http://www.plosbiology.org/article/info%3Adoi%2F10.1371%2Fjournal.pbio.1001908#s5

b) *Published Peer Review*

Please note while forming your response, if your article is accepted, you may have the opportunity to make the peer review history publicly available. The record will include editor decision letters (with reviews) and your responses to reviewer comments. If eligible, we will contact you to opt in or out. Please see here for more details:

https://blogs.plos.org/plos/2019/05/plos-journals-now-open-for-published-peer-review/

c) *Blot and Gel Data Policy*

Please provide the original, uncropped and minimally adjusted images supporting all blot and gel results reported in an article's figures or Supporting Information files. We will require these files before a manuscript can be accepted so please prepare them now, if you have not already uploaded them. Please carefully read our guidelines for how to prepare and upload this data: https://journals.plos.org/plosbiology/s/figures#loc-blot-and-gel-reporting-requirements

d) *Protocols deposition*

To enhance the reproducibility of your results, we recommend that if applicable you deposit your laboratory protocols in protocols.io, where a protocol can be assigned its own identifier (DOI) such that it can be cited independently in the future. For instructions see: https://journals.plos.org/plosbiology/s/submission-guidelines#loc-materials-and-methods

Thank you again for your submission to our journal. We hope that our editorial process has been constructive thus far, and we welcome your feedback at any time. Please don't hesitate to contact us if you have any questions or comments.

Sincerely,

Ines

--

Ines Alvarez-Garcia, PhD

Senior Editor

PLOS Biology

ialvarez-garcia@plos.org

------------------------------------

Reviewers' discussion

Rev. 1:

I would support the publication, though my minor concern still remains as I did not find the control data after reading the long PDF file:

Minor Concerns

1. The study identifies 35 candidate proteins in the supernatant of PA14-infected worms using mass spectrometry, narrowing these to five neuronal secreted proteins based on SignalP-5.0 and CeNGEN data. However, the manuscript does not report whether proteins from control conditions (e.g., OP50-treated or M9 buffer) were similarly analyzed to confirm specificity to PA14 infection. Providing a comparative proteomic profile of control supernatants would strengthen the claim that NSIF-1 (TAG-293) is specifically induced by PA14. The authors should include mass spectrometrydata from control conditions or explicitly state why such controls were not feasible.

Rev. 2:

The authors have done a very nice job responding to the 2nd set of critiques while under review at EMBO J. Reviewer 3's comments bring up one critical point that had been bothering me and can be addressed with one experiment that needs to be performed before publication. In the clarification of the timecourse, the authors argue that their data indicate that infection disrupts intact cuticles based on growing L4 animals on PA14 and imaging DPY-7::GFP and 0, 3, 6, 12, and 24 hours later. While their results are consistent with this model it still doesn't rule out an effect on building the cuticle that takes time to manifest. The authors should rule the confounding variable of molting by taking dpy-7::GFP young adults or L4+1 day animals and placing them on PA14 and imaging 0, 3, 6, 12, and 24 hours later. If they see the same results then it would definitively support their model and is a straightforward experiment to perform. Once that experiment is performed and the minor corrections listed below are addressed this manuscript will be suitable for publication in PLOS Biology.

Minor points

1.The authors should add citations for the strains obtained from the CGC. Some of these are missing from the reference section. Go through the strains in that section on the CGC site and be sure to add the appropriate citations.

2.The tissue-specific knock-out methods need to be removed since those experiments are no longer in the paper.

Rev. 4:

Summary:

In this manuscript, the authors discover that PA14 infection disrupts cuticle structure and hypodermal lysosome function in C. elegans. They implicate nsif-1 as the mediator of this process by demonstrating that it is required for the deleterious effects of PA14 on cuticle formation and lysosomal function. The authors then demonstrate that nsif-1 also functions in the intestine to regulate pmk-1-dependent immune response to PA14. While the identification of a neuronal-hypodermal host defense pathway is significant, the paper has many scientific and experimental deficiencies that need to be addressed before publication. In addition, the writing of this paper indicates a lack of attention to detail that needs to be addressed. For example, in the strain list, some genotypes are listed that are not presented in the paper at all; the figure legends are overly concise and do not accurately describe the data; and the methods sections frequently lack experimental detail.

Major issues:

The evidence that NSIF-1 secreted from ASI/ASJ and functions in the hypodermis presented in the paper is not complete. While the data presented indicate that neuronal-expressed NSIF-1 likely functions nonautonomously in the hypodermis, additional work is needed for the authors to conclude that NSIF-1 from ASI/ASJ is secreted to and functions in the hypodermis.

1)Demonstrate that fluorescently tagged NSIF-1 expressed only in ASI or ASJ is detected in the hypodermis and its nuclei, and that this is abolished when the SP of NSIF-1 is removed.

2)Demonstrate that NSIF-1 expressed only in ASI or ASJ rescues the cuticle and hypodermal lysosomal phenotypes of the nsif-1 mutant after PA14 infection and that this rescue is abolished when the SP of NSIF-1 is removed.

3)Show that the expression from other tissues (muscle or intestine) does not rescue the nsif-1 mutant phenotypes when infected with PA14.

4) Demonstrate that NSIF-1 expressed in the hypodermis rescues the nsif-1 mutant phenotypes when infected with PA14.

Issues in specific figures:

EV1B: How are "head" and "tail" defined? Landmarks? Size and position of a box?

S3: Fold change for each gene in each biological replicate should be plotted.

2A,2F and 2I: How are the locations of the 35umx25um boxes determined. How do you ensure that they are in the same place in every worm?

2G and 2J: Because heat shock is used to induce the expression of the NUC-1 reporters, more than one biological replicate should be performed.

2G, 2J, and 2M: Why are dots for values from individual worms not plotted as they are in 2B,2D, and 2E?

3C: Why were L1s used for the proteomics when all other experiments were performed on animals 24 hours post L4?

3C: How is this data normalized?

2F: Is this data the same as one of the plots in S7 with the overexpression strain removed? If not, where are the additional biological replicates for the survival of WT and nsif-1 mutant after infection?

S7: What promoter is used for the expression of the full-length nsif-1 here? Are these transgenic animals expressing extrachromosomal arrays, or is this integrated?

S7: Does overexpression of nsif-1 cause any changes to the cuticle structure or the hypodermal lysosomes?

2G and S8: Colocalization of the nsif-1 translational reporter with a neuronal-specific reporter should be performed, and images of a whole animal should be shown. This is particularly relevant because rgef-1 is used for neuronal expression later in the paper, but from the images presented, it is not clear how widespread nsif-1 is expressed in neurons.

From the pictures presented, it is not possible to tell if the nsif-1 translational reporter is not expressed in the hypodermis. Again, colocalization with a hypodermal reporter and whole worm images would make this clear.

4A and 4B: What p-value and fold change cut-off were used to determine differentially expressed genes in the nsif-1 mutant

4C: No statistics are presented here or for the RNA seq, so it is not appropriate to say these genes are "significantly upregulated".

S17: Why were L1s used for the RNA-seq when all other experiments were performed on animals 24 hours post L4?

EV5A and 5B: Does hypodermal expression of NSIF-1::GFP rescue the mutant phenotypes? The rationale for removing the SP from hypodermally expressed NSIF-1:GFP is unclear. Just because elt-3-regulated genes are deregulated in nsif-1 mutants does not mean that NSIF-1 must function in the nucleus. Does NSIF-1 have an NLS? How could it get diverted from the secretory pathways to the nucleus?

5B and 5C: How was effective knockdown of nsif-1 verified?

S14 and S15: How was the efficacy of ASI/ASJ ablation confirmed?

6A: From the images, it looks like ELT-2:GFP is more highly expressed in the nsif-1 mutant, but not that the localization has changed. This can be simply tested by qRT-PCR to quantify the level of endogenously expressed elt-3 mRNA.

# Author comment: Author response to Decision Letter 2

**DOI:** 10.1371/journal.pbio.3004016.r003

15 Jul 2026

Attachment Submitted filename: 02-Response-PLOS B.pdf

# Peer review: Decision Letter 2

**DOI:** 10.1371/journal.pbio.3004016.r004

**Authors:** Ines Alvarez-Garcia

10 Sep 2026

Dear Dr Qi,

Thank you for the submission of your revised Research Article entitled “Pathogen subversion of neuro-epidermal signaling impairs lysosomal function to disrupt collagen homeostasis in C. elegans” for publication in PLOS Biology. On behalf of my colleagues and the Academic Editor, Mark Alkema, I am delighted to let you know that we can in principle accept your manuscript for publication, provided you address any remaining formatting and reporting issues. These will be detailed in an email you should receive within 2-3 business days from our colleagues in the journal operations team; no action is required from you until then. Please note that we will not be able to formally accept your manuscript and schedule it for publication until you have completed any requested changes.

Please take a minute to log into Editorial Manager at http://www.editorialmanager.com/pbiology/, click the "Update My Information" link at the top of the page, and update your user information to ensure an efficient production process.

PRESS

We frequently collaborate with press offices. If your institution or institutions have a press office, please notify them about your upcoming paper at this point, to enable them to help maximise its impact. If the press office is planning to promote your findings, we would be grateful if they could coordinate with biologypress@plos.org. If you have previously opted in to the early version process, we ask that you notify us immediately of any press plans so that we may opt out on your behalf.

We also ask that you take this opportunity to read our Embargo Policy regarding the discussion, promotion and media coverage of work that is yet to be published by PLOS. As your manuscript is not yet published, it is bound by the conditions of our Embargo Policy. Please be aware that this policy is in place both to ensure that any press coverage of your article is fully substantiated and to provide a direct link between such coverage and the published work. For full details of our Embargo Policy, please visit http://www.plos.org/about/media-inquiries/embargo-policy/.

Many congratulations and thanks again for choosing PLOS Biology for publication and supporting Open Access publishing. We look forward to publishing your study.

Sincerely,

Ines

--

Ines Alvarez-Garcia, PhD

Senior Editor

PLOS Biology

ialvarez-garcia@plos.org
